# Whole-body fluorescence cryotomography identifies a fast-acting, high-contrast, durable contrast agent for fluorescence-guided surgery

**DOI:** 10.7150/thno.100802

**Published:** 2024-10-07

**Authors:** Augustino V. Scorzo, Brook K. Byrd, Caleb Y. Kwon, Rendall R. Strawbridge, Kimberley S. Samkoe, P. Jack Hoopes, Keith D. Paulsen, David W. Roberts, Scott C. Davis

**Affiliations:** 1 Thayer School of Engineering, Dartmouth College, Hanover, New Hampshire, USA.; 2 Department of Surgery, Geisel School of Medicine, Dartmouth College, Hanover, New Hampshire, USA.; 3 Dartmouth Cancer Center, Dartmouth-Hitchcock Medical Center, Lebanon, New Hampshire, USA.

**Keywords:** fluorescence-guided surgery, molecular imaging, neurosurgery, contrast agents, magnetic resonance imaging

## Abstract

Imaging of tumor-specific fluorescent contrast agents to guide tumor removal has been shown to improve outcomes and is now standard practice for some neurosurgical procedures. However, many agents require administration hours before surgery, a practical challenge, and may exhibit inconsistent concordance with contrast-enhanced MRI (CE-MRI), the current standard for diagnosing and guiding glioma removal. A fluorescent agent that accurately marks tumor shortly after administration and is otherwise similar to CE-MRI would help overcome these shortcomings.

**Methods:** We used whole-body 3-D fluorescence cryo-imaging and co-registered CE-MRI volumes to evaluate several fluorescent contrast agent candidates for diagnostic performance and concordance with CE-MRI. Mice with brain tumors were administered a cocktail of fluorescent agent candidates and a MRI contrast agent, and then imaged with MRI and fluorescence cryo-imaging at several timepoints after administration. The high-resolution 3-D cryo-imaging volumes of the fluorescent agents were used to determine diagnostic performance metrics and correlation with CE-MRI.

**Results:** While all agents showed positive metrics, one agent, tetramethylrhodamine conjugated to a small polyethylene glycol chain (TMR-PEG1k), outperformed the others, exhibiting minimal normal brain signal, high tumor-to-background-ratio, diagnostic accuracy, and cross-correlation to CE-MRI at all post-administration timepoints (10-90 min) and tumor lines examined.

**Conclusion:** These favorable properties establish TMR-PEG1k as a promising candidate for surgical guidance.

## Introduction

Surgical resection is a primary treatment for most solid tumors, and complete removal of tumor tissue increases the probability of a long-term positive outcome 1-4. Yet, in many surgical settings, difficulty in locating and distinguishing tumor from normal tissue remains a persistent challenge resulting in high rates of incomplete resection 5-9. To address this challenge, a wide array of strategies are available or under development, including probe-based measurements for tissue diagnosis, novel intraoperative imaging approaches, pathological analysis, and navigation using pre-surgical imaging 7,10-12. Although these efforts have had an impact on the field, incomplete tumor removal remains a significant problem.

A substantial portion of the innovation in surgical guidance has been focused on brain tumor resection. Management of this disease, and high grade gliomas in particular, is highly-dependent on the use of contrast-enhanced magnetic resonance imaging (CE-MRI), which has become the standard reference for diagnosis, surgical planning, intraoperative navigation, and post-operative assessment 5-7,11-16. CE-MRI scans report on the distribution of an MRI-sensitive contrast agent (typically based on gadolinium) shortly after the agent is administered. These agents are thought to be excluded from normal tissue by the blood brain barrier (BBB), while accumulating in tumors with abnormal, leaky vasculature with a compromised BBB 17. This differential uptake often gives rise to high tumor-to-background contrast in the MRI images. For surgical navigation, these CE-MRI images are acquired before surgery and registered to the patient on the operating table, enabling the tracking of surgical tools directly on the MRI volumes in real-time. Although routinely used and effective in surgical guidance, the reliance on pre-operative static scans results in increasing registration errors due to tissue changes that occur as the surgery progresses 11,12,18-21.

Fluorescence-guided surgery (FGS) is an alternative or adjunct strategy that aims to provide real-time tumor visualization directly from the tissue 22-37. The approach involves administering a fluorescent contrast agent designed to accumulate in or otherwise target tumors and using a fluorescence imaging system during surgery to visualize the agent distribution. Seminal work in the early 2000's led to the FDA-approval of aminolevulinic acid induced protoporphyrin IX (ALA-PpIX) fluorescence guidance for high-grade glioma resection in 2017 which is now standard practice at many medical centers 38-40. A large and growing body of work has shown that administering indocyanine green (ICG), an untargeted fluorescent contrast agent cleared for some indications, 24-48 h before surgery (termed “second window ICG” or SWIG imaging) produces high tumor contrast in brain and lung tumors 34,35,41-46. Additionally, several targeted agents which consist of a fluorophore conjugated to a moiety targeting an overexpressed receptor are in various stages of clinical development 47-50. Although many of these agents have been shown to produce high tumor-to-background tissue uptake, the spatial distribution of each is often unique, requiring new ways of interpreting the images, and many do not consistently recapitulate the accumulation observed with CE-MRI. They also almost universally require administering the agent several hours or even days prior to surgery to achieve optimal tumor contrast 26,47-49, which complicates clinical workflow and, in some cases, becomes impractical for patients traveling long distances for surgery. Thus, a fluorescent contrast agent that matches the distribution of an MRI contrast agent and provides high tumor contrast shortly after administration, similarly to CE-MRI, would alleviate this challenge and enable on-demand use during surgery. It would also provide image information that is already familiar to the surgical community.

In this context, the use of PEGylation to alter the behavior of therapeutic 51-57 or contrast agents 58,59 can be informative. Attaching PEG molecules of varying length and/or structure has been used to alter the kinetics of therapeutic agents 51,52 and/or prevent neurotoxic drugs from crossing the BBB 60-63. Applying the same logic, we previously hypothesized that using a fluorescent dye modified with a small PEG chain could confer many of the favorable properties observed with CE-MRI. Specifically, we sought an agent with a PEG chain small enough to retain relatively fast kinetics yet large enough to prevent ready passage through the normal BBB. In a small pilot study, we showed that fluorescein isothiocyanate (FITC) conjugated to a 550 Da mPEG chain improved tumor-to-background contrast (TBR) compared to FITC alone in mouse glioma models. This improvement in contrast was achieved entirely by reducing agent accumulation in normal brain tissue, even shortly after administration 64.

These encouraging results prompted the more extensive and systematic study of multiple candidate agents reported herein. Specifically, we used three dimensional (3-D) whole-body fluorescence cryo-imaging and co-registered CE-MRI to evaluate several candidate contrast agents at multiple timepoints in animal models. Whole animal cryo-imaging enabled agents to be assessed at high resolution in 3-D throughout the entire brain, and permitted direct comparison to co-registered CE-MRI images in the same animals. We considered four non-targeted fluorescent agents, two of which consisted of the same fluorophore conjugated to a 550 Da mPEG chain or a 1 kDa mPEG chain. We also included ICG, since it is a cleared, nontargeted agent showing promise in several tumor types, and a carboxylated form of LI-COR IRDye800CW, a non-targeted form of a commonly used near-infrared (NIR) fluorophore. The agents were co-administered with an MRI contrast agent into animals with orthotopic brain tumors, imaged using CE-MRI and then processed with whole animal fluorescence cryo-imaging. From the resulting high-resolution 3-D volumes, we assessed tumor and normal tissue uptake, tumor-to-background ratio, contrast-to-noise ratio, diagnostic performance using receiver operator curve analysis, and correlation to CE-MRI acquired at different times after administration. Based on these metrics, agents were compared to one another and to CE-MRI to identify, and a lead candidate was identified.

## Results

**Figure 1:**
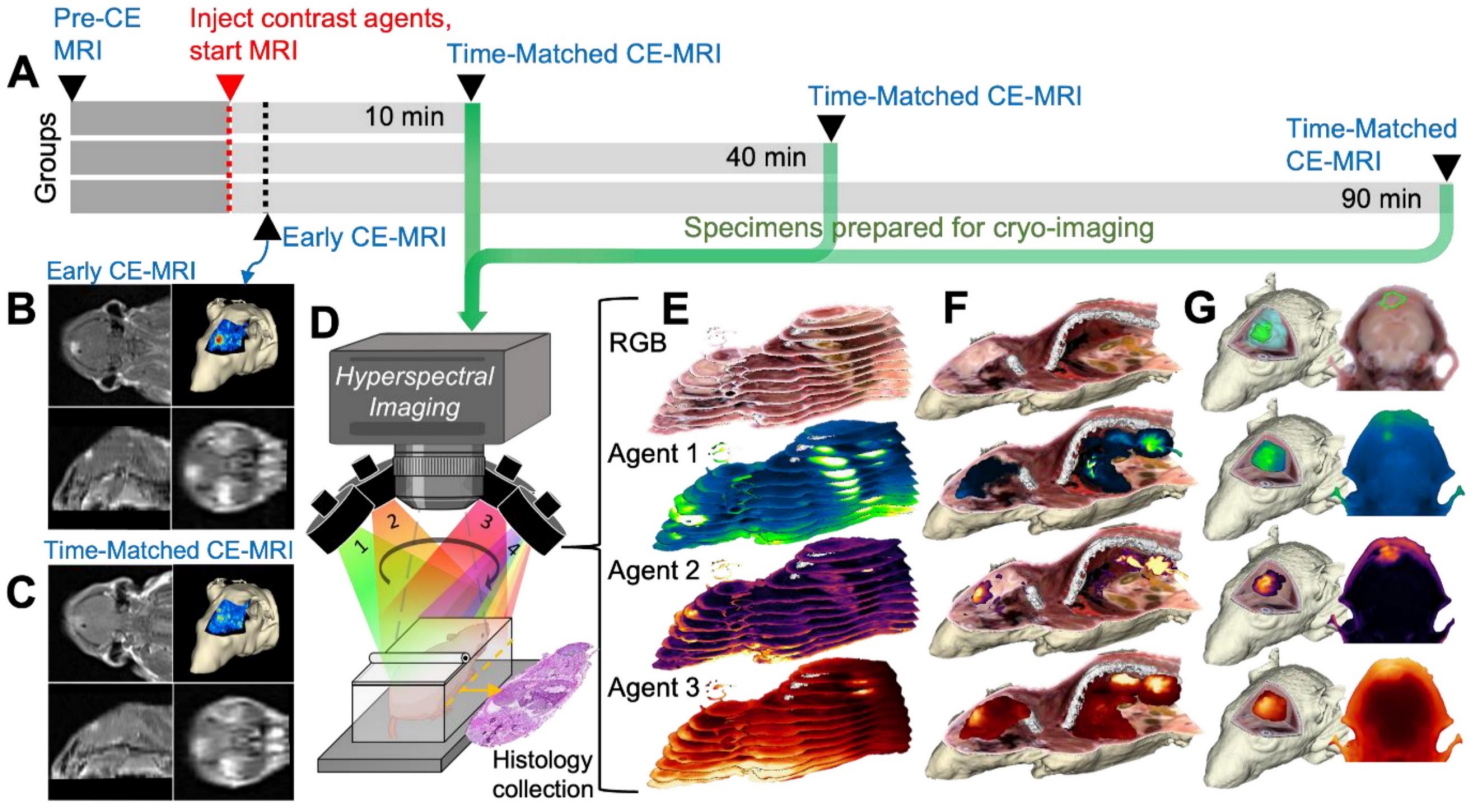
** Image acquisition process for CE-MRI and whole-body cryo-imaging of candidate fluorescent contrast agents.** (**A**) Experimental timeline. (**B**) For each animal, a T1-weighted MRI volume was acquired immediately after administration of the contrast agent cocktail, termed “Early CE-MRI”. (**C**) Another T1-weighted volume was acquired immediately before euthanasia at the specific evaluation time (10, 40 or 90 min after contrast agent administration), here termed “T.M. CE-MRI”. The specimen was then prepared for cryo-imaging. (**D**) Illustration of the hyperspectral cryo-imaging instrument which images the specimen block under sequential illumination of multiple sources during sectioning. (**E**) Representative image slices (12 of approximately 200 shown) from each channel illustrate how the image volume was acquired. (**F**) A rendered RGB volume with overlays of agent fluorescence plotted as maximum intensity projections (MIPs). (**G**) Renderings and selected 2-D images of just the head. The top row shows surfaces of the tumor and normal brain regions.

Whole-body multi-channel fluorescence cryo-imaging and co-registered CE-MRI were used to evaluate the 3-D spatial distribution of candidate fluorescent contrast agents at high resolution, and to compare agent distributions to one another and to conventional CE-MRI. Animals on study were randomized into one of three timepoint groups (10, 40 and 90 min) and followed the timeline described in Figure 1A. Specifically, anesthetized mice were placed in a conical tube which was then positioned in a specialized rodent MRI coil in a Siemens Prisma 3T scanner. Prior to contrast agent administration, pre-CE baseline T1-weighted (T1W) and T2-weighted (T2W) MRIs were acquired. Then, a solution consisting of a combination of fluorescent contrast agent candidates and a conventional MRI contrast agent was administered into the tail vein. T1W MRI scanning began immediately after agent administration to acquire the “Early CE-MRI” (Figure 1B) image stacks and was repeated at the designated study time for that animal to acquire the Time-Matched CE-MRI, here termed “T.M. CE-MRI” (Figure 1C). As soon as the T.M. CE-MRI concluded, the animal was euthanized and, without disturbing the position of the animal, the conical tube was filled with optimal cutting temperature (OCT) compound and frozen in preparation for whole animal cryo-imaging.

A schematic of the hyperspectral cryo-imaging technique is shown in Figure 1D. The instrument automatically sections large frozen specimens and acquires multichannel, hyperspectral images after each section is removed. The imaging process is accomplished by sequentially illuminating light sources (in this case, a broadband white light source and multiple fluorescence excitation sources), and for each source, acquiring multiple images at different wavebands using a liquid crystal tunable filter. Thus, the full data stack consists of a light spectrum at each pixel, for each source and each slice (Figure 1E). The processing procedure produced registered volumes of (1) the biodistribution of spectrally-distinct fluorescent contrast agents and (2) white light RGB volumes, which can be rendered in 3-D and overlaid, as depicted in Figure 1F-G. These volumes were then co-registered to the Early and Time-Matched MRI volumes for comparison between fluorescent and MRI contrast agents.

### All candidate agents exhibited elevated tumor contrast metrics, but TMR-PEG1k produced high, durable contrast within minutes of administration

**Figure 2:**
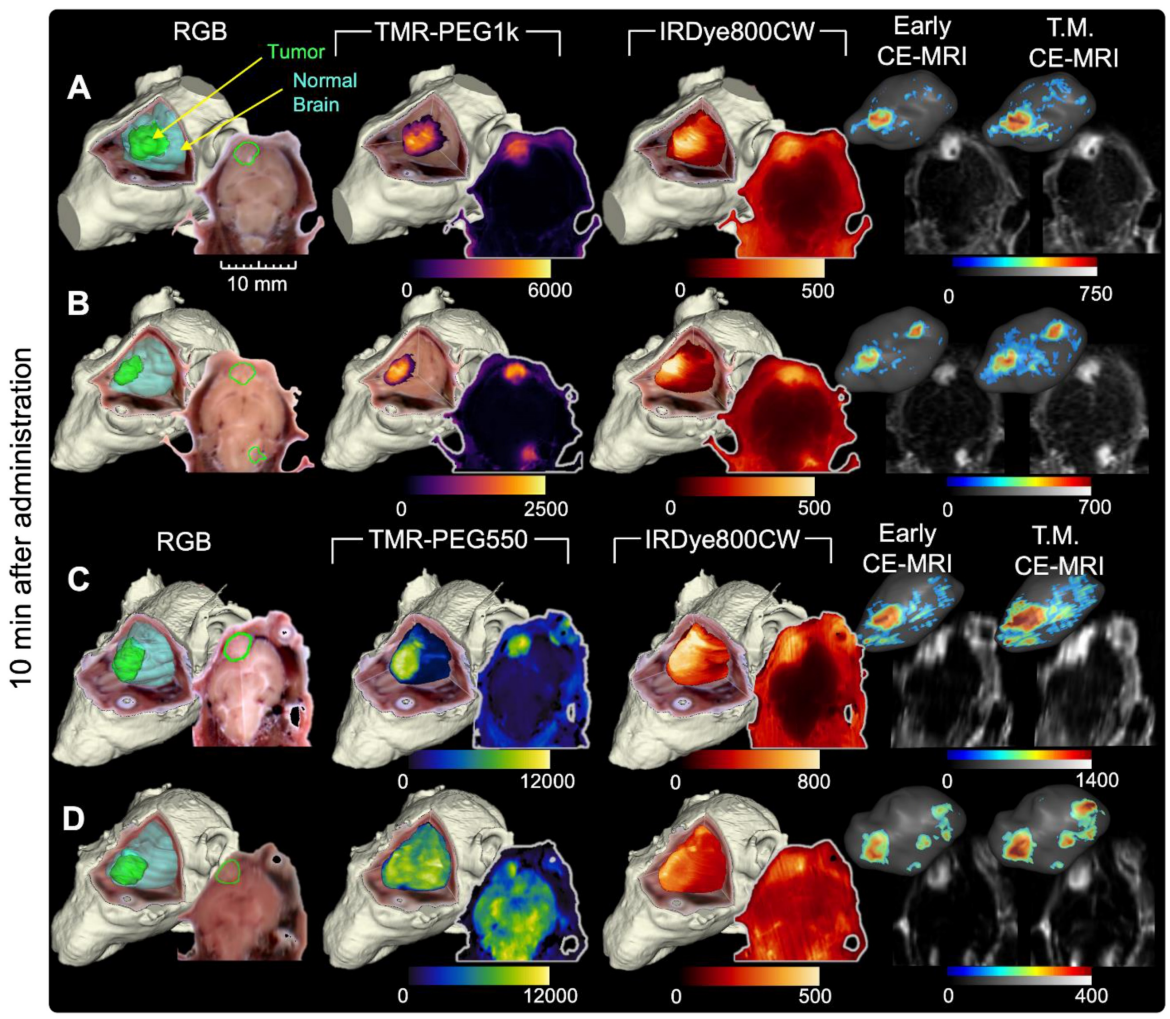
** Representative cryo-imaging and CE-MRI image volumes of mice with U251 tumors 10 min after agent administration.** Each row shows data for one animal. (**A, B**) Images of animals administered TMR-PEG1k and IRDye800CW. Columns from left to right show the RGB rendering with tumor and normal brain surfaces, TMR-PEG1k, IRDye800CW, Early CE-MRI and T.M. CE-MRI. (**C**, **D**) Images of animals administered TMR-PEG550 and IRDye800CW with an analogous column arrangement as described for (**A**) and (**B**). Units for fluorescence volumes are relative fluorescence units (RFU). The 10 mm scale bar applies to all 2-D images shown. No minimum threshold values were applied to the 2-D images. The fluorescence volume renderings were displayed as a MIP with an intensity threshold set to twice that of a naïve brain for that fluorescence channel. The 3-D renderings for the CE-MRI brain volumes were visualized with a signal intensity threshold of 4 times the noise floor.

**Figure 3:**
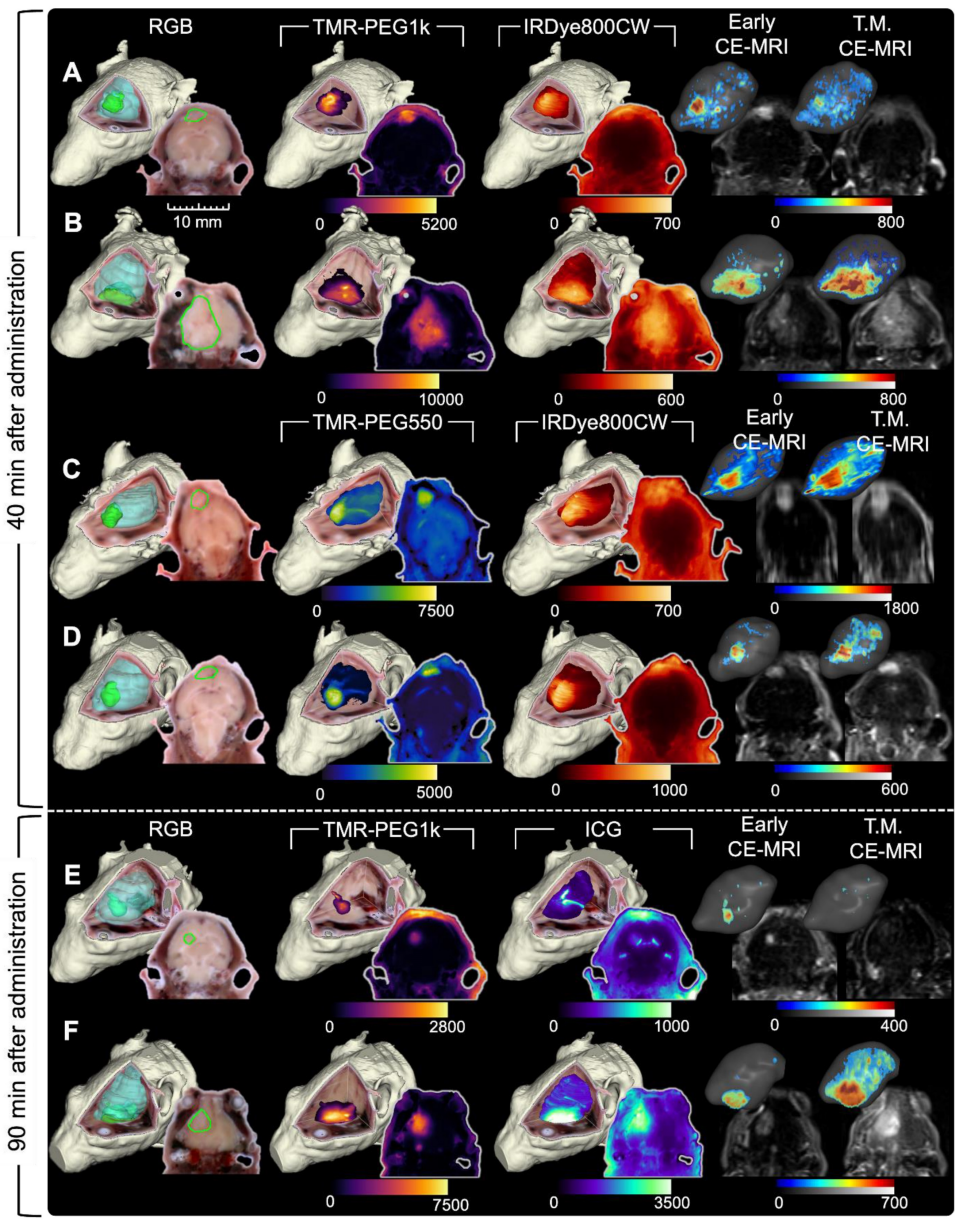
** Representative cryo-imaging and CE-MRI image volumes of mice with U251 tumors 40 and 90 min after agent administration.** Same structure as presented in Figure 2, each row shows data for one animal. (**A-D**) Show images of animals 40 min after agent administration. In (**A**) and (**B**), columns from left to right show the RGB rendering with tumor and normal brain surfaces, TMR-PEG1k, IRDye800CW, Early CE-MRI and T.M. CE-MRI. (**C**) and (**D**) shows images of animals administered TMR-PEG550 and IRDye800CW with an analogous column arrangement to (**A**) and (**B**). (**E**) and (**F**) show images of animals 90 min after administration of TMR-PEG1k and ICG. Units for fluorescence volumes are relative fluorescence units (RFU). Thresholding and units are identical to those used in Figure 2.

Figures 2 and 3 show representative cryo-imaging and MRI volumes for a subset of animals evaluated at 10 min (Figure 2), and 40 and 90 min (Figure 3) after fluorescent and MRI contrast agent administration. Each row represents an individual animal while the columns provide images and renderings of different contrast agents. Specifically, the first column shows a 3-D rendering of the RGB cryo-imaging stack overlaid with surfaces outlining the true tumor and normal brain regions (green and blue respectively); the second and third columns show the same RGB rendering overlaid with renderings of the fluorescence signal from a candidate contrast agent (TMR-PEG550, TMR-PEG1k, IRDye800CW or ICG); and the fourth and fifth columns show renderings of the Early and T.M. CE-MRI images. Each rendering is accompanied by a 2-D image slice selected from the corresponding co-registered cryo-imaging and MRI image stacks. For 3-D visualization, a minimum fluorescence signal threshold of twice the signal measured in naïve brain was applied. For 3-D CE-MRI visualizations, a minimum signal threshold of 4 times the noise floor was applied. No thresholding was applied to the 2-D slice images to show the entire range of values. Data for all remaining animals with U251 tumors are provided in supplementary Figure S1-S2.

Images for animals administered the TMR-PEG1k and IRDye800CW optical agents are shown in Figure 2A-B while Figure 2C-D provides images for animals administered TMR-PEG550 and IRDye800CW (all 10 min after administration). Qualitative inspection of these images reveals elevated fluorescence signal in tumor regions compared to normal brain in most cases for all fluorescent agents evaluated; however, notable differences in spatial uptake patterns occurred between agents. TMR-PEG1k appears to accumulate primarily in the tumor region with very little signal observed in normal brain tissue. Tumor boundaries seem sharp and subjectively concordant with the true tumor boundaries. TMR-PEG550, the PEGylated agent with a smaller PEG chain, produced higher signal intensity in tumor regions, but also showed higher uptake in normal brain tissues which varied widely between animals compared to TMR-PEG1k. For example, the signal intensity in the normal brain of the animal depicted in Figure 2D was similar to the intensity observed in the tumor, indicating widespread non-specific accumulation. Additionally, we observed uptake in ventricular structures with TMR-PEG550 (Figure 2C and S1G), a distribution not observed with TMR-PEG1k. In the IRDye800CW channel, the tumor region was observed to be higher than normal brain for all animals shown, though the normal brain signal was relatively high compared to TMR-PEG1k and a subset of TMR-PEG550 animals.

The Early and T.M. CE-MRI images (right-most two columns) were both acquired min after administration, within the time frame most-commonly used in the clinic. The uptake patterns look similar between these times and show sharp definition between tumor and normal brain tissues. Examination of Figure 2 suggests that of the three optical agents evaluated, TMR-PEG1k most closely resembles the CE-MRI images at this timepoint, and the qualitative similarity between these agents is notable.

Figure 3 has an identical structure to Figure 2 but shows data for a representative subset of animals evaluated 40 and 90 min after agent administration. These images show similar patterns to those observed in the 10 min group (Figure 2). Specifically, TMR-PEG1k (Figure 3A,B,E and F) continued to display high tumor signal, very low normal brain signal, and relatively sharp boundaries at both 40 and 90 min. TMR-PEG550, only evaluated at the 40 min timepoint (Figure 3C-D), also showed relatively high tumor contrast and sharp boundaries, but higher and more variable normal tissue uptake than the TMR-PEG1k agent. The IRDye800CW dye, also only evaluated at 40 min (Figure 3A-D), was present in tumor at elevated levels, but also produced signal in the background, similar to the results observed at the 10 min timepoint. ICG, which is known to have slow kinetics due to its binding to blood proteins, was evaluated at the 90 min timepoint and showed elevated tumor uptake in most cases, although signal in normal brain and ventricle regions also appeared elevated.

As in Figure 2, Early CE-MRI shows sharp tumor delineation, yet at later time-matched points (40 and 90 min), the CE-MRI signal appears reduced and/or dispersed in adjacent normal brain tissues in some animals. For example, the T.M. CE-MRI in Figure 3E, assessed at 90 min after administration, does not appear to show a clear tumor region which was previously visible in the Early CE-MRI. Notably, this reduction was not observed in all animals. In general, TMR-PEG1k images appear similar to Early CE-MRI images, but while the CE-MRI signal seems to fade and disperse over time, the fluorescent agent retains its sharp tumor demarcation.

**Figure 4:**
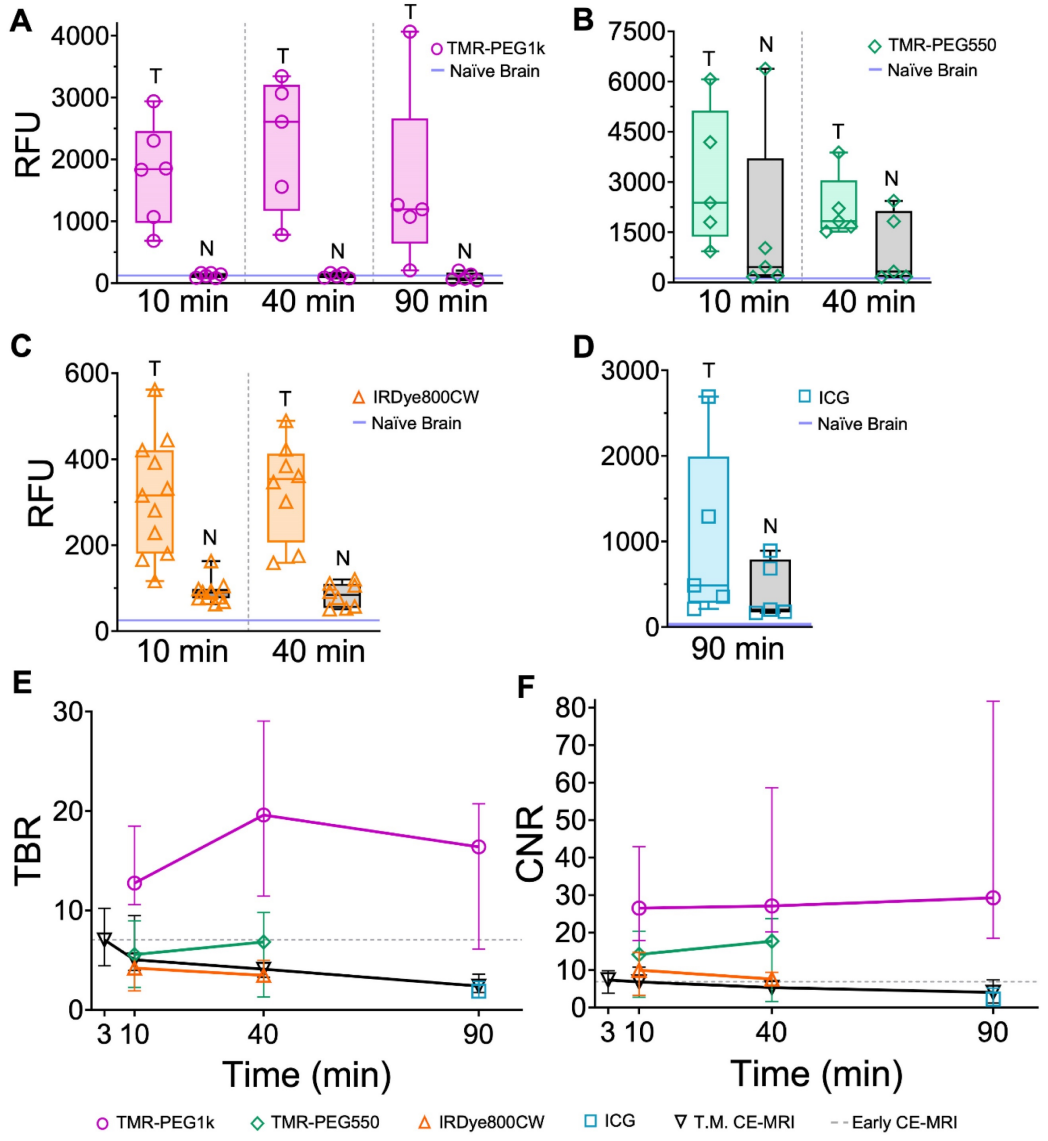
** Fluorescent contrast agent uptake and contrast evaluation in whole brains with U251 tumors.** (**A-D**) shows mean fluorescence intensity values of normal brain (N) and tumor tissues (T) for each fluorescent contrast agent presented as a box and whisker plot for (**A**) TMR-PEG1k, (**B**) TMR-PEG550, (**C**) IRDye800CW and (**D**) ICG. Each dot represents the mean intensity for an individual animal and the blue line indicates the value for a naïve brain from that fluorescence imaging channel. (**E**) Tumor-to-background ratio (TBR) for all contrast agents, including CE-MRI, at each timepoint represented as the median and interquartile range between animals (IQR). (**F**) Contrast-to-noise ratio (CNR) for all contrast agents at each timepoint represented as a median and IQR. The grey dotted line starting at 3 min and extending across the plot of (**E**) and (**F**) represents the median TBR and CNR for the Early CE-MRI acquisition.

Quantitative analysis of the imaging results confirms the qualitative observations. First, we examined the mean values in tumor and normal tissue for all agents at all timepoints. Figure 4A shows these values plotted for TMR-PEG1k. The blue line just above the y-axis represents mean normal brain values for noninjected naïve animals. Inspection of this graph indicates that this dye accumulated in tumor tissue with negligible uptake in the normal brain at all timepoints. Mean tumor values were >10 times that of background at the 10 min timepoint, peaked at 40 min and were lowest at 90 min, though still produced a large differential compared to normal tissue at this later timepoint. Confirming the qualitative evaluation of the images, mean signal values of TMR-PEG550 (Figure 4B) in tumor were higher than those observed using TMR-PEG1k; however, normal brain values were also substantially higher. Specifically, the median normal tissue values for TMR-PEG550 were 3.8 and 2.9 times those observed with TMR-PEG1k at 10 and 40 min, respectively. Figure 4C shows tumor and normal brain values of IRDye800CW at the 10 and 40 min timepoints and Figure 4D shows the same for ICG at the 90 min timepoint, again with the blue line indicating naïve brain background signal. For both dyes, median tumor values were elevated above background at all timepoints, but background signal was relatively high.

Next, we evaluated TBR for all agents and timepoints. Figure 4E shows the median TBR and interquartile range for all fluorescent contrast agents and CE-MRI. The early CE-MRI value of 7 is shown at a 3 min timepoint and depicted as a dotted line across all timepoints, for reference. All agents produced median tumor contrast >1 at all timepoints; however, performance varied significantly across agents. TMR-PEG1k produced high TBR contrast (= 13) after just 10 min, and remained high with a TBR = 20 after 40 min and 16 at the 90 min timepoints. These TMR-PEG1k TBR values were substantially higher at all timepoints than the highest contrast value of any other agent. TMR-PEG550 produced TBR values of 6 at 10 min which increased to 7 at 40 min, achieving similar contrast performance to the Early CE-MRI; IRDye800CW provided TBR values of 4 and 3 at 10 and 40 min, respectively, while ICG produced TBR = 2 at 90 min. CE-MRI contrast was highest directly after administration at the Early CE-MRI timepoint (TBR = 7) and decreased to TBR = 5, 4 and 2 at 10, 40 and 90 min, respectively.

Comparing contrast-to-noise ratio (CNR) values for all agents and timepoints further illustrates the difference in performance between the agents (Figure 4F). As in Figure 4E, the Early CE-MRI data are presented as a line from the time of acquisition (3 min after administration) through all timepoints. TMR-PEG1k produced high and consistent CNR at all timepoints, with CNR = 27, 27, and 29 at the 10, 40 and 90 min timepoints, respectively. These values are substantially higher than the agent with the next-highest CNR values, TMR-PEG550, which produced CNR = 14 and 18 for the 10 and 40 min timepoints, respectively. IRDye800CW provided CNR values of 10 and 8 (10 and 40 min, respectively) and ICG produced a CNR of 2 at 90 min The CE-MRI CNR peaked at the Early CE-MRI timepoint (CNR = 7) and steadily decreased over time (CNR = 7, 5, and 4 at 10, 40 and 90 min timepoints, respectively).

Results from a statistical comparison of TBR and CNR are provided in Table 1 and confirm TMR-PEG1k's superior performance compared to other agents. Specifically, TMR-PEG1k produced significantly higher TBR contrast than any other fluorescent contrast agent tested at all timepoints. It also provided significantly higher CNR values than the other agents, with the exception of TMR-PEG550 at the 40 min timepoint. At this timepoint, the difference in CNR between TMR-PEG1k and TMR-PEG550 was not significant (p = 0.05). TMR-PEG550 did not outperform IRDye800CW at the 10 or 40 min timepoints. A paired statistical analysis performed between contrast agents co-administered to the same animal also showed significantly higher TBR and CNR produced by TMR-PEG1k vs. other agents (Table S1).

### TMR-PEG1k exhibited superior diagnostic performance and a high correlation with CE-MRI

**Figure 5:**
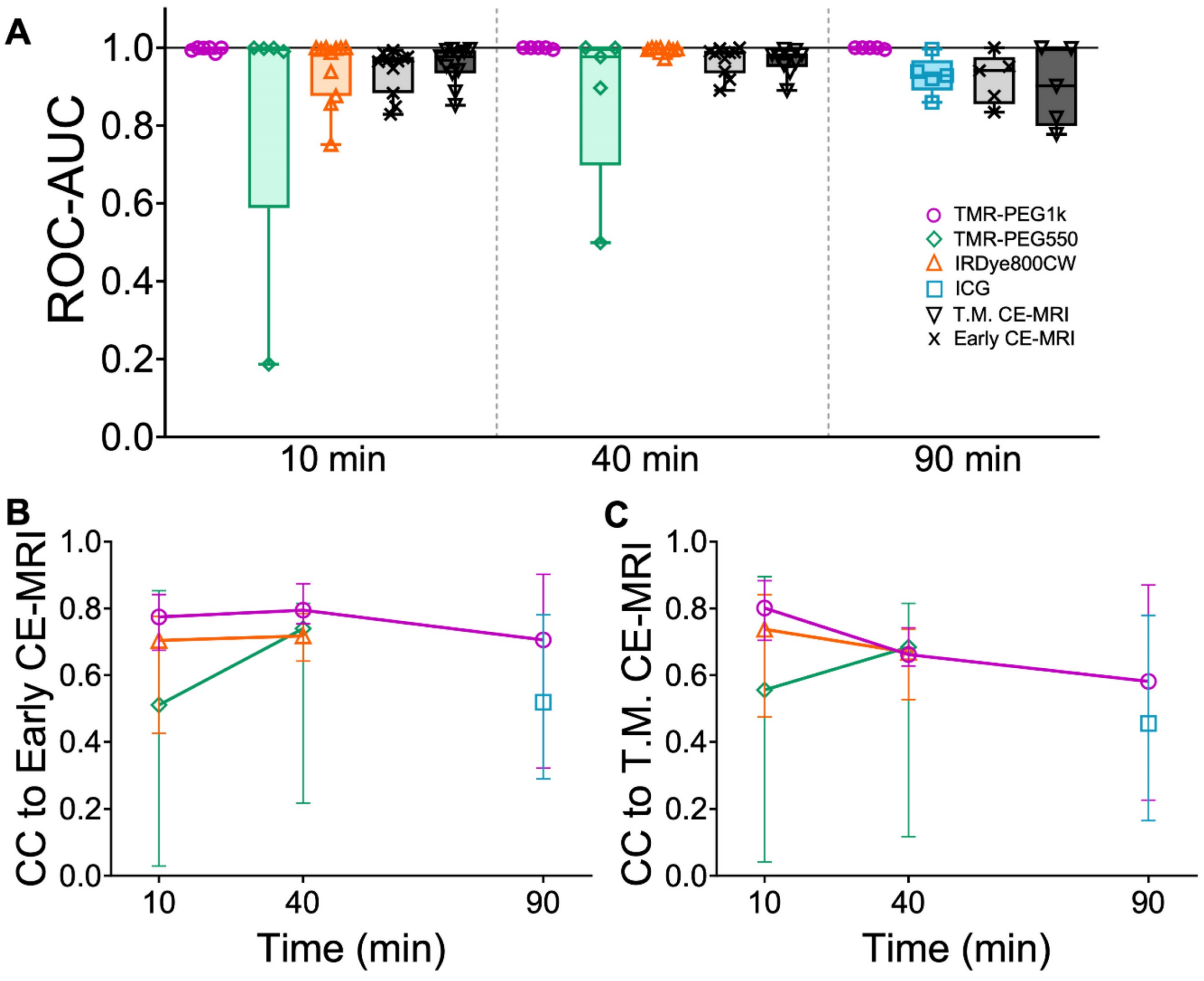
** Diagnostic performance of contrast agents and cross-correlation between fluorescent contrast agents and CE-MRI in U251 tumors.** (**A**) Area under the receiver operating characteristic curve (ROC-AUC) values at each timepoint for all contrast agents and all animals. Normalized cross-correlation (CC) with Early CE-MRI (**B**) and T.M. CE-MRI (**C**) for each fluorescent contrast agent at every timepoint, represented as a median with IQR.

To evaluate diagnostic performance of the contrast agents, area under the receiver operating characteristic curve (ROC-AUC) was calculated at each timepoint using the volumetric images for all agents. Figure 5A shows the ROC-AUC for all contrast agents and each timepoint displayed as box plots. TMR-PEG1k achieved a median ROC-AUC of 0.999, 1.00, and 0.999 at 10, 40 and 90 min with all values for each animal above 0.985. Most other agents also showed robust diagnostic performance, with median ROC-AUCs above 0.895; however, higher inter-subject variability was observed for these agents, with AUCs for individual animals decreasing to the 0.5-0.6 range in some cases. TMR-PEG550, for example, showed high median performance (ROC-AUC = 0.998 and 0.977 at 10 and 40 min, respectively), but an inability to diagnose tumors in a handful of animals (ROC-AUC ≤ 0.5). ROC-AUC values for IRDye800CW at 10 and 40 min were 0.996 and 0.998 respectively, and though variability occurred at the earliest timepoint with individual animals having ROC-AUCs as low as 0.751, they were consistently high at 40 min. The median ROC-AUC for ICG was 0.928 and values as low as 0.860 were observed. The Early CE-MRI acquired immediately after administration was combined from all three timepoint groups providing a median ROC-AUC value of 0.967. ROC-AUC values for T.M. CE-MRI were similar to the Early CE-MRI values, with the lowest median value of 0.901 observed at the 90 min timepoint.

To evaluate the similarity between fluorescent contrast agents and CE-MRI uptake, we assessed the normalized cross-correlation (CC) between each fluorescent contrast agent image stack and the corresponding CE-MRI images in the whole brain region of the same animals. Figure 5B shows the CC comparison of all fluorescent contrast agents to Early CE-MRI (CC to Early CE-MRI) and Figure 5C shows CC for fluorescent contrast agents vs. Time-Matched CE-MRI plotted as the median with interquartile range. When compared to Early CE-MRI images, TMR-PEG1k produced the highest median CC at each timepoint, with all values above 0.7 and a peak at 40 min. TMR-PEG550 produced relatively low values (CC = 0.5) at 10 min, but higher values at the 40 min timepoint (CC = 0.74). IRDye800CW exhibited values of 0.70 and 0.72 at 40 and 90 min respectively, and ICG showed the lowest values at 90 min (CC = 0.52). Similar trends were observed when fluorescent contrast agents were compared to Time-Matched CE-MRI, with the exception of the TMR-PEG1k channel, which showed a steady decline in CC with the T.M. CE-MRI over time (from 0.80 at 10 min to 0.58 at 90 min). Notably, TMR-PEG1k at all timepoints had high CC to the CE-MRI images that are most commonly used in clinical practice (Early CE-MRI), yet showed lower CC to CE-MRI images acquired at later timepoints as the MRI contrast agent appeared to disperse from the tumor region.

### Evaluation in a second tumor line with a GFP reporter confirmed TMR-PEG1k's diagnostic performance and correlation to CE-MRI

**Figure 6:**
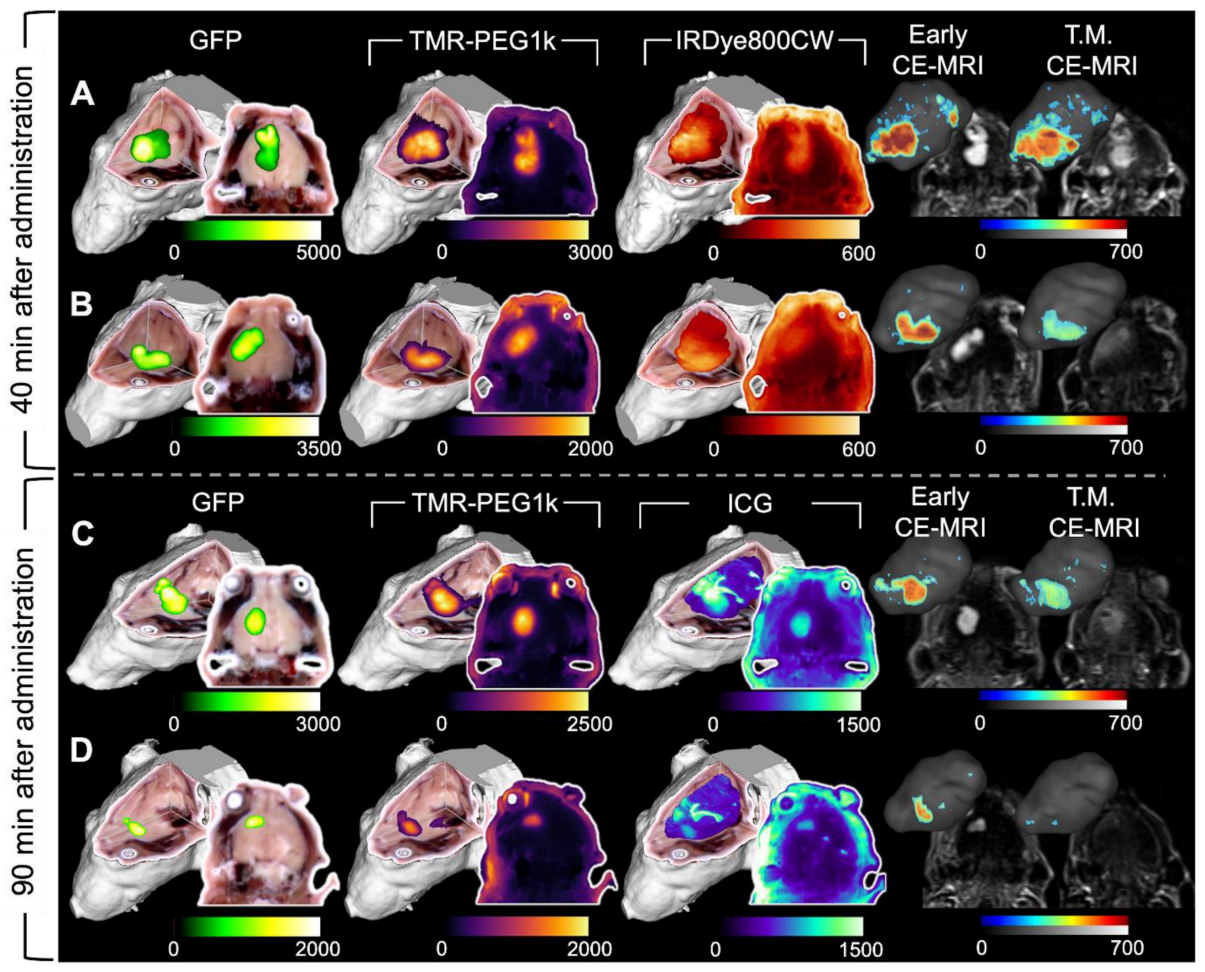
** Representative cryo-imaging and CE-MRI image volumes of mice with U87-GFP tumors 40 and 90 min after agent administration.** Same structure as presented in Figure 2 and 3, each row represents one animal. (**A-B**) Show images of animals 40 min after administration. The columns from left to right show the GFP rendering, TMR-PEG1k, IRDye800CW, Early CE-MRI and T.M. CE-MRI. (**C-D**) Show images of animals 90 min after administration of TMR-PEG1k and ICG with an analogous column arrangement as described for A-B. Thresholding and units are identical to Figure 2 and 3.

TMR-PEG1k was evaluated further using a tumor line that expressed green fluorescent protein (GFP) 40 and 90 min. after agent administration. In these experiments, we also included IRDye800CW or ICG for comparison. Representative cryo-imaging and MRI volumes for these animals are shown in Figures 6 and S3 following the structure of Figure 2 with the exception of the first column, which now shows the cryo-imaged GFP volumes. Inspection of Figure 6 reveals behavior similar to that observed in the U251 tumors for all agents tested. TMR-PEG1k appeared to accumulate specifically in tumor regions and low signal was observed in the normal brain tissue while IRDye800CW and ICG exhibited relatively high normal tissue signal. Notably, TMR-PEG1k also appeared to closely match the distribution of GFP fluorescence in all animals.

**Figure 7:**
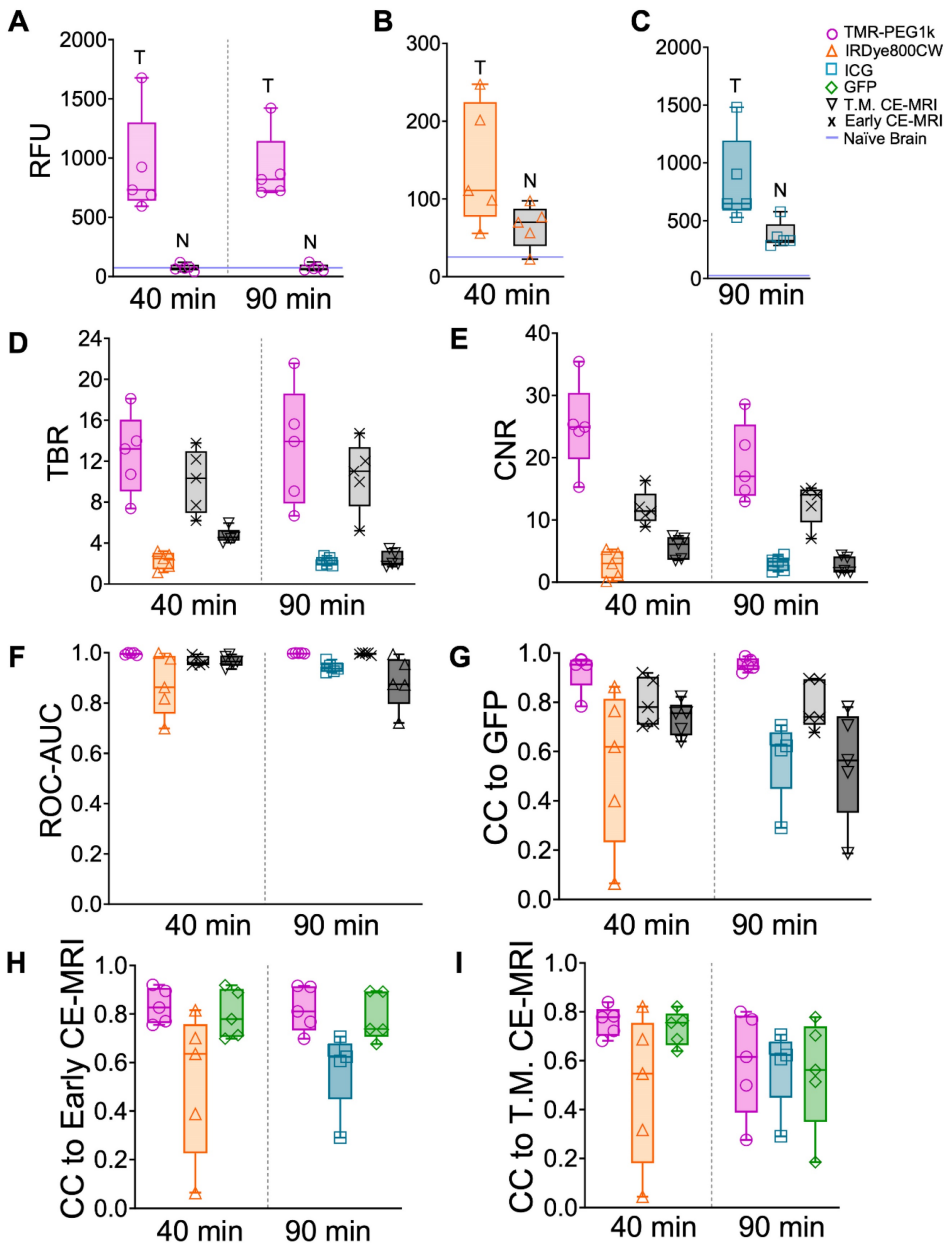
** Fluorescent contrast agent uptake, contrast evaluation and diagnostic evaluation in whole brains of mice with U87-GFP tumors.** (**A-C**) shows mean fluorescence intensity values of brain and tumor tissues for each fluorescent contrast agent presented as a box and whisker plot for (**A**) TMR-PEG1k, (**B**) IRDye800CW and (**C**) ICG. Each dot represents the mean intensity for an individual animal and the blue line indicates the value for a naïve brain from that fluorescence imaging channel. (**D**) Tumor-to-background (TBR) ratio for all contrast agents, including CE-MRI, at each timepoint represented as a boxplot with and interquartile range (IQR). Contrast-to-noise (CNR) ratio for all contrast agents at each timepoint represented as a boxplot and IQR. (**F**) Area under the receiver operating characteristic curve (ROC-AUC) values at each timepoint for all contrast agents and all animals. Normalized cross-correlation (CC) with GFP (**G**), Early CE-MRI (**H**) and T.M. CE-MRI (**I**) for each fluorescent contrast agent at every timepoint, represented as a median with IQR.

Using GFP-defined tumor volumes, mean intensity values in tumor and normal brain, TBR, CNR, and ROC-AUC were computed for these animals and plotted in Figure 7A-F. These results broadly confirm the findings obtained in the U251 tumor line models. Mean TBR values for TMR-PEG1k were > 13 at both timepoints, largely due to the low signal in the normal brain, while the presence of IRDye800CW and ICG in normal brain resulted in lower TBR values. CNR and ROC-AUC metrics were also strikingly similar to those observed in the U251 animals, with TMR-PEG1k showing high CNR and near-perfect ROC values with very little variance. Results from a statistical comparison of TBR and CNR values between agents are provided in Table 2.

The normalized cross-correlation computed between the 3-D distribution of each agent and GFP fluorescence, Early CE-MRI, and T.M. CE-MRI are plotted in Figure 7G-I, respectively. TMR-PEG1k was highly correlated to GFP, with a mean CC = 0.95 at both timepoints. This correlation result was even higher than the CC between CE-MRI and GFP. The CC values between TMR-PEG1k and CE-MRI were similar to those observed in the U251 tumor line animals, with a mean CC > 0.81 for both 40 and 90 min timepoints when compared to Early CE-MRI. This CC decreased when compared to the T.M. CE-MRI. The mean CC values for IRDye800CW and ICG compared to either GFP or CE-MRI were significantly lower and showed much higher variance as compared to the TMR-PEG1k results.

### Co-registered pathology confirms tumor identification using RGB and GFP imaging channels

**Figure 8:**
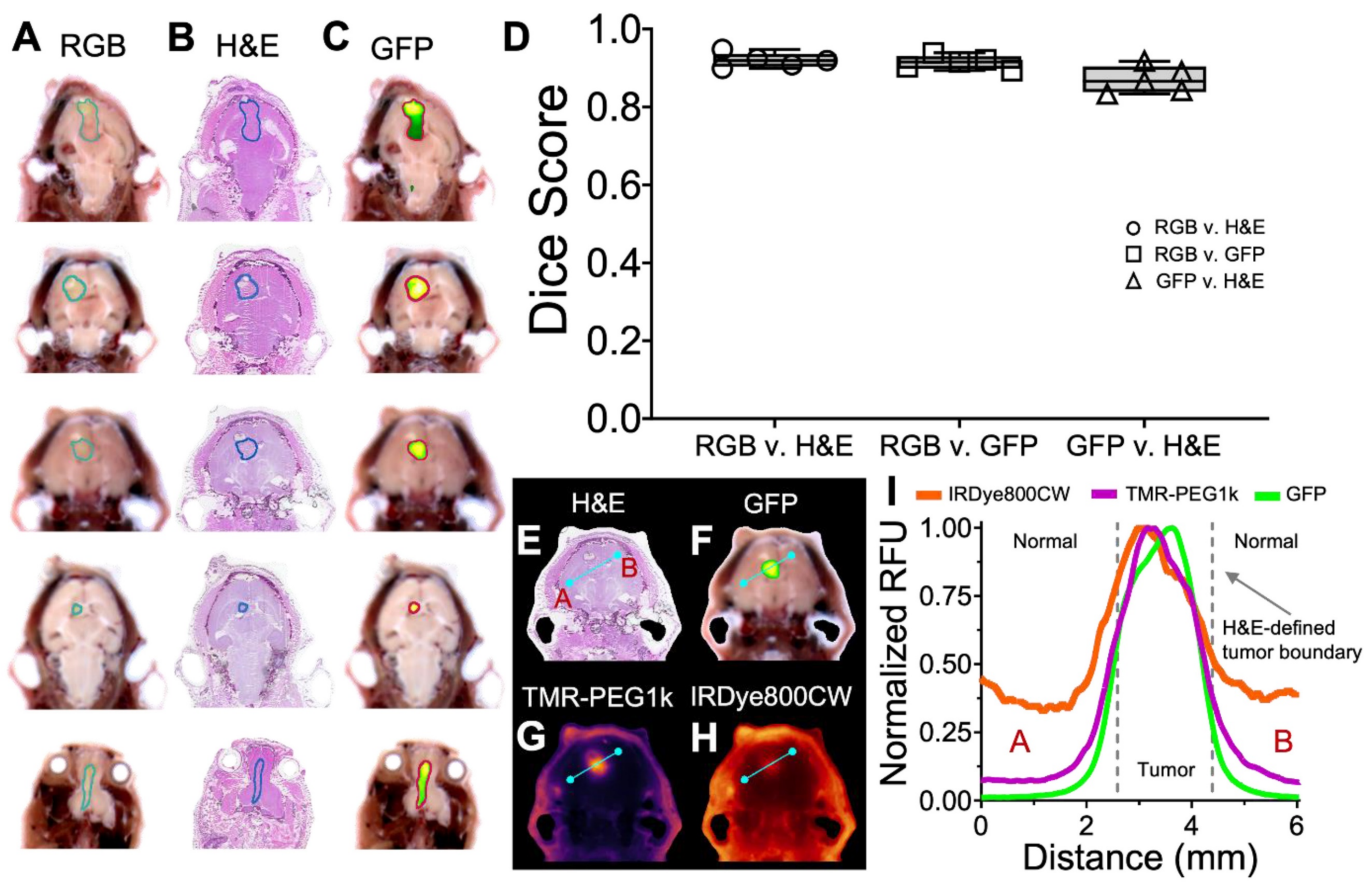
** Confirming cryo-imaging results with co-registered H&E.** (**A-C**) Co-registered RGB, H&E and GFP fluorescence images of one section from 5 study animals, with tumor ROI overlays. (**D**) Dice similarity scores between ROI's determined from RGB, H&E and GFP fluorescence images, plotted as medians with interquartile ranges. (**E-H**) Co-registered H&E, GFP and contrast agent fluorescence for one section of one animal. (**I**) Normalized fluorescence intensity profiles of GFP and contrast agent fluorescence.

To confirm that the RGB and GFP image channels accurately identify tumor and normal tissue in this study, we evaluated the similarity between these channels and co-registered H&E in five animals from the U87-GFP tumor groups. Tumor ROIs were drawn independently on a 2-D slice from each of the three image channels (H&E, RGB, and GFP), as shown in Figure 8A-C, and the Dice score calculated between each. These scores (Figure 8D) indicate high similarity between each of the groups, confirming that the RGB and GFP channels are suitable surrogates for independently identifying tumor.

Examination of the contrast agent channels in these same slices enables comparison between GFP, H&E and the contrast agents across the brain, and reveals the difference between TMR-PEG1k and other agents around the tumor border. RGB, H&E, GFP, and contrast agent image sets with corresponding normalized cross-sectional profiles are provided in Figure 8E-I and S4-S5. Inspection of these results indicates that TMR-PEG1k closely mimics GFP, both of which exhibit low normal brain signal and relatively sharp features at the H&E-defined tumor border. IRDye800CW and ICG, on the other hand, produced higher background signal and smaller gradients at the tumor borders.

## Discussion

In this study, we sought to identify fluorescent contrast agents that provide high tumor contrast and diagnostic performance with a short administration-to-imaging time, similar to MRI contrast agents commonly used in MRI for tumor demarcation. Specifically, we investigated whether PEGylation could be used to improve the kinetic behavior of fluorescent contrast agents to achieve these favorable properties. Using whole animal fluorescence cryo-imaging and co-registered MRI, we evaluated four candidate contrast agents, two of which were conjugated to small PEG chains, at multiple timepoints in two glioblastoma mouse models. Using the resulting 3-D image volumes, we assessed contrast metrics, diagnostic performance and correlation to CE-MRI for each agent.

The results herein largely confirm our central hypothesis, that a PEGylated non-targeted fluorescent contrast agent can provide high contrast and diagnostic performance shortly after administration, similar to CE-MRI. We found that all four contrast agents provided elevated tumor-to-background contrast (median TBR >3 in most cases) and high diagnostic performance (median ROC-AUC > 0.86) at all timepoints; yet significant differences in performance were found between agents. Specifically, TMR-PEG1k, a fluorophore conjugated to a 1 kDa mPEG chain, outperformed all other agents in every metric. This agent produced a median TBR of 13 just 10 min after administration, and high TBR persisted throughout the time course investigated (up to 90 min), reaching a peak (TBR = 20) at 40 min. These TBR values are among the highest reported in the literature for both untargeted and targeted fluorescent contrast agents, even at long incubation times 47-49,65. TMR-PEG1k also produced superior diagnostic performance metrics compared to the other agents, providing median ROC-AUC values >0.99 at all timepoints with minimal intra-subject variance, suggesting that this agent could be a highly effective tumor marker.

Closer examination of the data suggests that the superior performance of TMR-PEG1k was driven largely by reduced agent accumulation in normal brain, not increased tumor uptake. Unlike the other agents, which exhibited fluorescence well above baseline in normal brain tissue, the median TMR-PEG1k fluorescence intensity observed in normal brain tissue was indistinguishable from that observed in brains of control animals at all time points, suggesting that the PEG1k version of the agent, with a total molecular weight of 1.5 kDa, was large enough to be excluded from crossing the BBB into the normal brain. This was not the case for the smaller TMR agent (TMR-PEG550, total molecular weight = 990 Da), which exhibited high inter-subject variability and overall elevated normal brain tissue accumulation. This behavior resulted in inconsistent contrast and diagnostic performance across subjects, and overall lower performance across all metrics compared to TMR-PEG1k. A reasonable explanation is that the smaller version of the agent was excluded from the normal BBB in some animals but was permitted to cross in others; although the factors that differentiate these two groups are unclear. The larger 1 kDa version of the molecule was far more consistent in its performance, and thus has emerged as the lead candidate for further advancement.

PEGylation of therapeutic and other agents has been used for a variety of purposes for decades 51-53,66,67, including to exclude potentially neurotoxic therapeutic agents from crossing the normal BBB 60-63. Here, we applied the same principle to dramatically increase tumor-to-background contrast for contrast-based diagnostic imaging during surgical guidance. Other efforts have examined PEGylated fluorescent agents for surgical guidance more broadly. Kang *et al.* reported on a comprehensive study of fluorophores conjugated to PEG molecules between 1k and 40 kDa at times ranging from 1 to 24 h after administration in flank tumor models, finding that optimal TBR was achieved with 20 kDa PEG chains 24 h after administration 58. Our study aimed to achieve high contrast after short administration time intervals in the specific case of brain imaging. Thus, we reasoned that choosing a molecular size that was small enough to provide relatively rapid kinetics yet large enough to be excluded by the BBB, similar to CE-MRI contrast agents, would achieve this aim. Our results indicated that this effect was achieved with the 1.5 kDa agent (TMR-PEG1k) and, to a lesser extent the 990 Da agent (TMR-PEG550). Although further tuning of the PEG size and structure may yield improvements in performance, the metrics reported herein are highly favorable and support further investigation of TMR-PEG1k.

An FGS agent that mimics the behavior of clinical CE-MRI would also provide information that is already familiar to many surgeons directly in the surgical field. Thus, evaluating the similarity of candidate fluorescent contrast agents to CE-MRI was a secondary objective. Here again, TMR-PEG1k outperformed the other agents. Using correlation metrics calculated between the 3-D cryo-imaging volumes and co-registered CE-MRI volumes of the whole brain, we found that TMR-PEG1k was highly correlated to CE-MRI acquired within minutes of administration, i.e., the sequence/timepoint typically used for diagnostic clinical imaging. Interestingly, the correlation remained high when comparing this Early CE-MRI acquisition to TMR-PEG1k acquired at all timepoints, suggesting that TMR-PEG1k maintains a relatively stable distribution up to 90 min after administration (the longest time evaluated) that closely mimics Early CE-MRI. The later “Time-Matched” CE-MRI images showed the MRI contrast agent dispersing throughout the normal tissue, reducing contrast and diagnostic performance metrics. These results suggest that an FGS agent that strictly mimics CE-MRI over time would actually be disadvantageous. In this context, the observation that TMR-PEG1k closely mimics Early CE-MRI at all timepoints but does not necessarily correlate with CE-MRI behavior over time is an unexpected, yet favorable result.

The high performance of TMR-PEG1k at such short times after administration, and the observed stability of the contrast produced by TMR-PEG1k over a long time period, would be highly advantageous and have the potential to ease existing constraints in deploying fluorescence guidance in clinical practice. Current FGS agents, and those in advanced clinical development, all require administering agents at least several hours, and up to a day, before surgery, which can be burdensome to clinical staff and patients alike. This is especially challenging for hospitals with large catchment areas to which patients travel for care. Once administered, contrast that is stable throughout the procedure would be ideal. Most glioma surgeries require between 3 and 4 hours from the time of incision, while complex surgeries can approach 6 hours. This study examined performance of TMR-PEG1k up to 90 minutes after administration, finding that the diagnostic performance was highly stable from the earliest, 10 min time point, through the latest, 90 minutes; however, we anticipate the contrast would remain stable well beyond this time point since rapid changes in distribution after long periods of stability for any agent are uncommon. Additionally, preliminary studies in our lab using flank tumor xenografts in mice (data not shown) suggest that contrast may be stable through at least 6 hours, though these results require validation. Finally, if contrast is shown to diminish significantly at later times during surgery, the rapid nature of the agent may enable re-administration as needed, which is a focus of future work.

The clinical standard for fluorescence guidance in brain tumor resection is imaging of 5-aminolevulinic acid (5-ALA)-induced protoporphyrin IX (PpIX), which was approved for use in high grade glioma in the United States in 2017. This strategy involves administered the pro-drug 5 -ALA, resulting in accumulation of the fluorophore PpIX in cells with high metabolic activity over the course of several hours. The approach has been proven to improve progression-free survival significantly, but also has well-known performance and practical shortcomings. Specifically, ALA-PpIX has been shown to have suboptimal negative predictive values (ranging from 0.26 to 0.66) 68-70, requires administration hours before surgery and does not always correlate with CE-MRI. TMR-PEG1k, or agents with similar properties would alleviate the administration time constraint and more closely approximate CE-MRI. Importantly, the latter property could expand the use of FGS to other brain-based tumor types for which ALA is not currently indicated, such as brain metastases. A comprehensive study to quantify the difference and potential complementarity between ALA-PpIX and TMR-PEG1k will be an important aspect of further development efforts.

In this study, we choose tetramethylrhodamine as the fluorescent label for the PEGylated contrast agents, which is a bright fluorophore in the visible regime. Although fluorescence from visible dyes is limited to superficial layers of tissue and is highly affected by the presence of blood, it also tends to provide sharp, high-resolution images of dye distribution which some surgeons may prefer. The use of NIR dyes, on the other hand, facilitates the detection of light from sub-surface structures; however, fluorescence from deeper layers produces a diffuse cloud of signal that may confound image interpretation. Importantly, the same PEG chains used herein can be conjugated to longer-wavelength NIR dyes and thus the use of visible, NIR, or both regimes can be evaluated directly for the specific indication. This evaluation is underway in our lab.

The whole-body/organ fluorescence cryo-imaging approach applied herein enabled high-resolution, 3-D assessment of agent biodistribution and direct comparison to co-registered MRI images. We believe this approach can serve as a model for assessing fluorescent contrast agents in preclinical development. A common approach for evaluating FGS agents involves acquiring 2-D images of fresh gross sections which provide a limited sample of the tissue volume and are difficult to co-register to other imaging modalities under investigation, such as MRI. The cryo-imaging approach described herein addresses those shortcomings and enables high-resolution assessment of agent performance throughout the tissue volume. The primary disadvantage of specimen cryo-imaging is the inability to evaluate multiple timepoints in the same animal. Thus, a combination of *in vivo* kinetic imaging for temporal information and 3-D cryo-imaging of specimens at a sampling of relevant timepoints would establish a complete assessment of agent distribution.

The results reported herein confirm our hypothesis and establish TMR-PEG1k as a compelling candidate for surgical guidance that can provide high diagnostic performance with a short administration time for a favorable imaging protocol. Although evaluated here in a glioma model, TMR-PEG1k, or similar pegylated agents, may be applicable to a broader set of cancer types, and assessment of uptake in other target organs is underway. The use of a novel high-fidelity 3-D imaging technique is an important aspect of the study that enabled reliable evaluation of candidate agents throughout the entire volume. These results support further development of TMR-PEG1k for fluorescence-guided surgery, and studies in a large animal tumor model are underway.

## Materials and Methods

### Experimental design

The study was designed to evaluate the three-dimensional biodistribution and diagnostic performance of fast-acting fluorescent contrast agent candidates for surgical guidance and compare the performance of these agents to a conventional MRI contrast agent in multiple tumor lines. A timeline of the experimental procedure is provided in Figure 1A. Specifically, mice with orthotopic brain tumor xenografts were administered a solution containing multiple spectrally-distinct fluorescent contrast agent candidates, as well as a MRI contrast agent. CE-MRI images were acquired shortly after administration to provide “Early CE-MRI” volumes commonly used in clinical surgical guidance, and again at a designated time, 10, 40, or 90 min after administration to provide an MRI volume Time-Matched to the cryo-imaging volumes, here termed “Time-Matched CE-MRI” or “T.M. CE-MRI”. Immediately after the final MRI acquisition, the animal was euthanized and, without repositioning, submerged in optimal cutting temperature compound (OCT) and frozen in preparation for whole-body cryo-imaging. Once frozen, the specimen was sliced at 100 μm sections and the fluorescent contrast agents imaged after every slice to produce high-resolution whole-body volumes of agent biodistribution. Table 3 provides a summary of the experimental groups.

For each animal, the CE-MRI volumes and corresponding, co-registered fluorescent contrast agent volumes were used to report agent uptake values for tumor and normal brain, tumor-to-background contrast (TBR), contrast-to-noise ratio (CNR), area-under-the-curve for receiver operator characteristic analysis (ROC-AUC), and cross-correlation (CC) between different agents. Each of these parameters were evaluated at each designated timepoint (10, 40 and 90 min after administration) and compared to each other as well as to the "Early CE-MRI” and “T.M. CE-MRI” images. For animals with tumor cells expressing the green fluorescent protein (GFP) reporter, these metrics were also compared to the 3-D cryo-imaging volumes of GFP.

### Animal models

All experiments were performed in accordance with protocols approved by the Institutional Animal Care and Use Committee at Dartmouth College. For the small animal mouse studies, female nude mice (Charles River Laboratories, Wilmington, MA, USA) between seven and ten weeks old were inoculated with 10^6^ U251 glioma cells or 10^6^ U87 glioma cells expressing GFP (Neuromics, Minneapolis, MN, USA) using a previously-described intracranial surgical procedure 71.

Prior to surgery, and 24 hours after the procedure, mice received subcutaneous ketoprofen dosed at 5 mg/kg. Immediately after surgery animals were placed on a chlorophyll-free diet (MP Biomedicals, Irvine, CA, USA) to minimize tissue autofluorescence. Tumor growth was monitored using CE-MRI until a tumor size of ~2 mm was reached, at which time animals were put on study, a process which took between 2 and 5 weeks. To ensure residual MRI contrast agent had cleared from the pre-study monitoring scans, a minimum of 24 hours elapsed before data acquisition scans.

### Fluorescent contrast agents

Four untargeted fluorescent contrast agents were evaluated:

1) TMR-PEG550: Tetramethylrhodamine conjugated to a 550 Da mPEG chain (Creative PEGWorks, Durham, NC, USA. Dosed at 42.1 nmol; peak absorbance/emission = 560/573 nm, total molecular weight: 991 Da).

2) TMR-PEG1k: Tetramethylrhodamine conjugated to a 1 kDa mPEG chain (same manufacturer, dose and fluorescent properties as listed above, total molecular weight: 1,480 Da).

3) IRDye800CW Carboxylate (LI-COR Biosciences, Lincoln, NE, USA, dosed at 1 nmol; maximum absorbance/emission = 774/789 nm, total molecular weight: 1,090 Da).

4) ICG: Indocyanine green (Chem-Impex International, Wood Dale, IL, USA, dosed at 317 nmol; maximum absorbance/emission = 790/814 nm, total molecular weight: 775 Da).

All agent stocks were prepared under sterile conditions. The ICG stock was prepared using a 1:1 mixture of water and phosphate buffered saline (PBS) no more than 3 hours before injection. The other three fluorescent contrast agents were prepared from aliquots previously reconstituted with PBS and kept at -20 °C for bolus preparation. Prior to use, contrast agent concentration was confirmed using absorption spectrometry.

### CE-MRI acquisition procedure

The data acquisition process was designed to record full MRI volumes before and at various timepoints after contrast agent administration, and corresponding multi-channel high-resolution cryo-imaging volumes of the same animals that could be co-registered to the MRI images. Since cryo-imaging must be completed after euthanasia, mice were randomized into one of three time-point groups; namely, 10, 40 and 90 min after administration prior to data acquisition. For all animals, an Early CE-MRI scan was acquired (immediately after administration of the contrast agent) as well as a CE-MRI scan at the designated time to match the cryo-imaging volumes (the “T.M. CE-MRI”), as detailed below.

In preparation for acquisition, a study animal was anesthetized with isoflurane (3% induction and 0.75-2% maintenance) and then positioned in a 50 mL falcon tube modified to permit a continuous flow of oxygen and isoflurane. The tube was placed in a custom rodent MRI radio frequency coil 72 and positioned in a Siemens Prisma 3.0T MRI scanner. Data acquisition began with pre-contrast MRI scanning followed by agent administration and MRI scanning until the designated study timepoint for that animal, at which point the animal was euthanized and prepared for cryo-imaging. Specifically, the scan sequence involved the following procedure:

1) Prior to contrast agent administration, T1-weighted (TR: 592 ms, TE: 25 ms, FOV: 68 mm, resolution: 0.3 mm x 0.3 mm x 0.8 mm and slice number = 25) and T2-weighted (TR: 2000 ms, TE: 200 ms, FOV: 84 mm, resolution: 0.3 mm x 0.3 mm x 0.3 mm and slice number: 80) images were acquired of the mouse head.

2) Mice were then administered a 200 µL MRI-fluorophore contrast agent cocktail via tail vein injection. This solution consisted of the gadolinium-based contrast agent for contrast enhanced MRI (Dotarem; Guerbet, Princeton, NJ, USA, dosed at 30,000 nmol, molecular weight: 754 Da) and a combination of fluorescent contrast agents under investigation at the doses listed in the fluorescent contrast agent section.

3) Immediately after administration of the contrast agent cocktail, another T1-weighted sequence was initiated, termed “Early CE-MRI” herein.

4) Another set of T1-weighted images, the “Time Matched CE-MRI” or “T.M. CE-MRI”, were acquired at the designated cohort times (10, 40 or 90 min). After imaging, the animal was immediately euthanized without being moved via CO_2_ inhalation. The falcon tube was then capped, filled with optimal cutting temperature (OCT) compound (Fisher Scientific, Pittsburgh, PA, USA), and frozen at -20 ° C in preparation for cryo-imaging.

Each frozen whole-body specimen was then mounted in the imaging cryomacrotome and processed to produce multi-channel volumes.

### Whole-body hyperspectral cryo-imaging

A detailed description of the whole-body hyperspectral fluorescence cryo-imaging system has been reported previously 73. Briefly, the system consists of an array of LED and laser light sources, and a two-camera hyperspectral imaging system mounted on a Leica CM3600 whole animal cryomacrotome for autonomous image acquisition and sectioning of a specimen block. In this study, up to four light sources were used: a 6500 K white light LED (Mightex, Toronto, ON, Canada) for RGB imaging, a 470 nm LED (Mightex, Toronto, ON, Canada) with a 475 nm short pass filter for GFP imaging, a 530 nm LED (Mightex, Toronto, ON, Canada) with a 550 nm short pass filter and a 760 nm laser (CrystaLaser LC, Reno, NV, USA). Light re-emitted from the fluorescence specimen was detected with a 500 mm focal distance objective lens (50.8 mm diameter, AR coated Achromatic Doublet, Thorlabs, Newton, NJ, USA) and then spectrally split into two channels using a 750 nm dichroic mirror (Thorlabs, Newton, NJ, USA). Light below above 750 nm passed through a motorized filter wheel containing long pass emission filters followed by a liquid crystal tunable filter (LCTF, Varispec, Woburn, MA, USA) before being detected with a scientific CMOS camera (PCO Edge 4.2, Bavaria, Germany). Light above 750 nm was directed through a 780 nm long pass filter before detection with a second CMOS camera.

Custom control software written in LabVIEW (National Instruments, Austin, TX, USA) coordinated automatic sectioning and imaging of the frozen specimen block. After each section, the specimen block paused to permit the imaging system to acquire images while sequencing through each light source and LCTF detection waveband, as illustrated in Figure 1B-C. This process was repeated automatically until the entire specimen was sectioned and imaged. In this study, each automated section was 100 µm thick. For each specimen, automated sectioning was paused at least once to manually collect 20 µm sections for histological staining. These sections were collected using Kawamoto's film method 74 which involves applying specialized cryofilm tape to the surface of the specimen block and manually peeling the tape as the block passed under the blade.

The resulting imaging data consisted of RGB and next-image corrected hyperspectral image stacks for each light source and slice (with the exception of the ICG and IRDye800CW channels which are not acquired through the LCTF), as depicted in Figure 1C. The image processing pipeline described previously was used to produce high resolution, co-registered, three-dimensional volumes of the following channels: white light RGB, GFP (for animals with GFP-expressing tumors), Tetramethylrhodamine fluorescence (for the TMR-PEG agents), and ICG or IRDye800CW fluorescence, as depicted in Figure 1F-G 73. For the fluorescence channels, the “Next-image correction” technique was used to compensate for fluorescence signal originating from structures below the slice being analyzed (“out-of-slice” fluorescence). This is an established technique 75,76 that subtracts an attenuated version of the image acquired after the slice has been removed. Once processed, the resulting volumes were visualized using 3D Slicer (https://www.slicer.org) 77. To minimize spectral overlap of TMR-PEG1k and GFP for the animals with GFP-expressing tumors, emission in the 470 nm channel was analyzed from 510 - 530 nm and the emission from the 530 nm channel was analyzed from 620 - 650 nm. Control animals that had not been administered contrast agents were imaged and processed in a similar manner to quantify naïve background brain fluorescence levels in each channel.

### Histopathology

Immediately after collecting the specimen on the cryofilm during whole animal cryo-imaging, tissue sections were stained with hematoxylin (Epredia Hematoxylin 7211, Fisher Scientific, Waltham, MA, USA) and eosin (Epredia Eosin-Y 7111, Fisher Scientific, Waltham, MA, USA) using a standard H&E staining procedure. Histological slides were scanned on the Odyssey M (LI-COR Biosciences, Lincoln, NE, USA) at 10 µm resolution and co-registered to the cryo-imaged volumes using a point-based rigid registration in 3D Slicer.

### MRI and cryo-image co-registration

Contrast-only CE-MRI volumes for each animal were computed by subtracting the pre-contrast agent T1W MRI volumes from the post-contrast agent images of the same animal. To compensate for any changes in animal position between the pre- and post-contrast agent administration images, these image stacks were first co-registered using a model-based registration of the eyes and brain. This process started by manually segmenting the eyes and brain of the pre- and post-contrast agent image volumes to create two 3D models that were then registered using the SlicerIGT module in 3D Slicer. This technique performs an iterative closest point method to minimize the root mean square error (RMSE) between the two models. With the two MRI volumes now in close alignment, an intensity-based registration was conducted using the General registration (BRAINS) module with the following parameters: sampling percentage = 30%, initialization = useCenterOfHeadAlign, degrees of freedom = 6, relaxation factor = 0.5, maximum step length = 0.05 and cost metric = normalized correlation. Target registration error was computed between 7 anatomical feature points within the brain of the pre- and post-contrast MRI volumes and confirmed to be < 500 µm. For each animal, two CE-MRI volumes were calculated: One using the T1W images acquired within min of the contrast agent administration (the “Early CE-MRI,” which is the timepoint commonly used for clinical CE-MRI) and one immediately before euthanasia at the specified 10, 40 or 90 min time (“T.M. CE-MRI”).

Next, the cryo-imaging volumes were co-registered to the MRI volumes, which was accomplished in a two-step process similar to the one described above. First, the eyes and brain in the RGB cryo-image volume were manually segmented and registered to the eyes and brain of the pre-contrast MRI volume using the SlicerIGT module. This procedure brought the two volumes into near alignment. Next, a point-based registration was performed between distinguishable features of the RGB and T2W MRI volumes. Specifically, 12-15 identifiable points (primarily ventricular structures) were manually selected throughout the brain in both the T2W MRI and RGB volumes. The registration was then completed using a rigid transformation of these point sets applied in SlicerIGT's fiducial registration wizard to achieve the MRI-to-RGB cryo-imaging registration. Because the fluorescence and RGB cryo-images are acquired with the same camera system, the resulting transformation was also applied to all fluorescence channels, ensuring that all cryo-imaging channels were registered to the MRI volumes.

### Image analysis

To establish tumor and normal tissue ground truth for analysis of mice with U251 tumors, the RGB volumes were manually segmented into tumor and normal brain ROIs in 3-D. To ensure tumor regions were selected independently, the segmentation was performed with the user blinded to the fluorescence and MRI volumes, and segmented tumor regions were confirmed with corresponding co-registered stained histopathology slices read by a trained pathologist. The cryo-imaged fluorescent volumes and ROIs were then resampled into MRI image space using 3D Slicer for quantitative analysis. For mice implanted with GFP-expressing tumor cells, tumors were segmented using the GFP fluorescence.

The volumetric ROIs were then applied to all fluorescence volumes, Early CE-MRI volumes, and T.M. CE-MRI volumes to compute the following parameters of interest for analysis:

1) Mean intensity values of the fluorescent contrast agent signal in tumor and normal brain

2) Tumor-to-background tissue contrast (TBR)



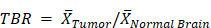



3) Contrast-to-noise ratio (CNR)



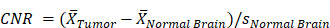



4) Area-under-the-curve from receiver operator characteristic analysis (ROC-AUC) using the perfcurve function in Matlab 78.

5) Normalized cross-correlation (CC) between each fluorescent contrast agent volume and the corresponding Early and T.M. CE-MRI volumes in each animal resulting in a CC-Early CE-MRI and CC-T.M. CE-MRI. Additionally, for GFP animals, CC between all contrast agent volumes (fluorescent CAs and the MRI contrast agent) and the corresponding GFP fluorescence volume was calculated resulting in CC-GFP.



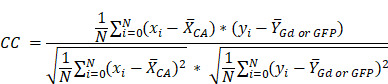



Where:


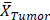
 Mean tumor value


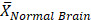
 Mean normal brain value


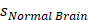
 Standard deviation of normal brain


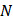
 Number of values


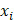
 Fluorescent contrast agent value


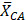
 Mean fluorescent contrast agent value


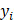
 MRI contrast agent (Gd) or GFP fluorescence value


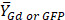
 Mean MRI contrast agent (Gd) or GFP fluorescence value

### Statistical analysis

Results are presented as medians with error bars reporting the interquartile range unless otherwise indicated. Before analysis, all data were tested for the normality condition using a Shapiro-Wilk test; α = 0.05. Additionally, variances between groups were tested for statistically significant differences using a Brown-Forsythe test; α = 0.05. After satisfying these two conditions, a one-way ANOVA was performed to assess significant differences between the fluorescent contrast agent means for TBR and CNR at 10 and 40 min timepoints separately. Following a significant result, Tukey's post-hoc analysis was conducted using a single pooled variance to compare all means within each metric's timepoint. A two-tailed paired t-test was performed for TBR and CNR respectively at 90 min to compare TMR-PEG1k and ICG in the same animal. The significance level was set to 0.05 for all of the analyses. To determine diagnostic ability, ROC-AUC values were generated using the perfcurve function in MATLAB which plots the true positive rate against the false positive rate as the classification threshold varies and computes the resulting area under the curve (AUC). Statistics were performed in Prism version 9 (GraphPad, San Diego, CA, USA). No data or outliers from the reported fluorescent contrast agents were omitted from the analysis in this study.

## Supplementary Material

Supplementary figures and table.

## Figures and Tables

**Table 1:** P-values for comparing TBR and CNR for different fluorescent contrast agents in U251 tumors.

**TBR Comparison**	**10 min**	**40 min**	**90 min**
TMR-PEG1k vs. TMR-PEG550	0.001^**^	0.007^**^	---
TMR-PEG1k vs. IRDye800CW	<0.0001^****^	0.001^**^	---
TMR-PEG1k vs. ICG	---	---	0.02^*^
TMR-PEG550 vs. IRDye800CW	0.51^ns^	0.88^ns^	---
			
**CNR Comparison**	**10 min**	**40 min**	**90 min**
TMR-PEG1k vs. TMR-PEG550	0.03^*^	0.05^ns^	---
TMR-PEG1k vs. IRDye800CW	0.002^**^	0.007^**^	---
TMR-PEG1k vs. ICG	---	---	0.04^*^
TMR-PEG550 vs. IRDye800CW	0.84^ns^	0.76^ns^	---

Note: ^****^p ≤ 0.0001, ^***^p ≤ 0.001, ^**^p ≤ 0.01, ^*^p ≤ 0.05, ^ns^p ≥ 0.05.

**Table 2:** P-values comparing TBR and CNR for different fluorescent contrast agents in U87-GFP tumors.

**TBR Comparison**	**40 min**	**90 min**
TMR-PEG1k vs. IRDye800CW	<0.0001^****^	---
TMR-PEG1k vs. ICG	---	0.0005^***^
		
**CNR Comparison**	**40 min**	**90 min**
TMR-PEG1k vs. IRDye800CW	<0.0001^****^	---
TMR-PEG1k vs. ICG	---	<0.0001^****^

Note: ^****^p ≤ 0.0001, ^***^p ≤ 0.001, ^**^p ≤ 0.01, ^*^p ≤ 0.05, ^ns^p ≥ 0.05.

**Table 3:** Summary of animal groups in the small animal study.

	Administration-to-imaging time	Tumor line	Fluorescent contrast agents	Number of animals
Group 1a	10 min	U251	TMR-PEG550 IRDye800CW Carbox.	5
Group 1b	10 min	U251	TMR-PEG1k IRDye800CW Carbox.	6
Group 2a	40 min	U251	TMR-PEG550 IRDye800CW Carbox.	5
Group 2b	40 min	U251	TMR-PEG1k IRDye800CW Carbox.	5
Group 2c	40 min	U87-GFP	TMR-PEG1k IRDye800CW Carbox.	5
Group 3a	90 min	U251	TMR-PEG1k Indocyanine Green	5
Group 3b	90 min	U87-GFP	TMR-PEG1k Indocyanine Green	5

## Abbreviations

2-D: two-dimensional
3-D: three-dimensional
ALA-PpIX: aminolevulinic acid induced protoporphyrin IX
ANOVA: analysis of variance
BBB: blood brain barrier
CA: contrast agent
CC: normalized cross-correlation
CE-MRI: contrast-enhanced MRI
CNR: contrast-to-noise ratio
FITC: fluorescein isothiocyanate
FGS: fluorescence-guided surgery
FOV: field of view
GFP: green fluorescent protein
Gd: gadolinium
H&E: hematoxylin and eosin
ICG: indocyanine green
IQR: interquartile range
LCTF: liquid crystal tunable filter
MRI: magnetic resonance imaging
NIR: near infrared
OCT: optimal cutting temperature compound
RFU: relative fluorescence units
ROC-AUC: area under the receiver operating characteristic curve
ROI: region of interest
SWIG: second window indocyanine green
T1W: T1-weighted MRI
T2W: T2-weighted
TBR: tumor-to-background ratio
TE: time to echo
TMR-PEG1k: tetramethylrhodamine conjugated to a 1 kDa methoxy polyethylene glycol chain
TR: time to repetition
