# Supplementary material for: Whole-body fluorescence cryotomography identifies a fast-acting, high-contrast, durable contrast agent for fluorescence-guided surgery
Source: Theranostics. 2024 Oct 7;14(17):6426–45. doi: 10.7150/thno.100802 (PMC11519800; doi:10.7150/thno.100802)
Supplement: Supplementary file 1 — Supplementary figures and table. [file thnov14p6426s1.pdf]

Supplementary Materials for

Whole-body fluorescence cryotomography identifies a fast-acting, high-contrast, durable contrast agent for fluorescence-guided surgery

Augustino V. Scorzo *et al.*

Corresponding author: Scott C. Davis. Email: Scott.C.Davis@dartmouth.edu

This file includes:

Figures S1 to S5
Table S1

Figure S1.

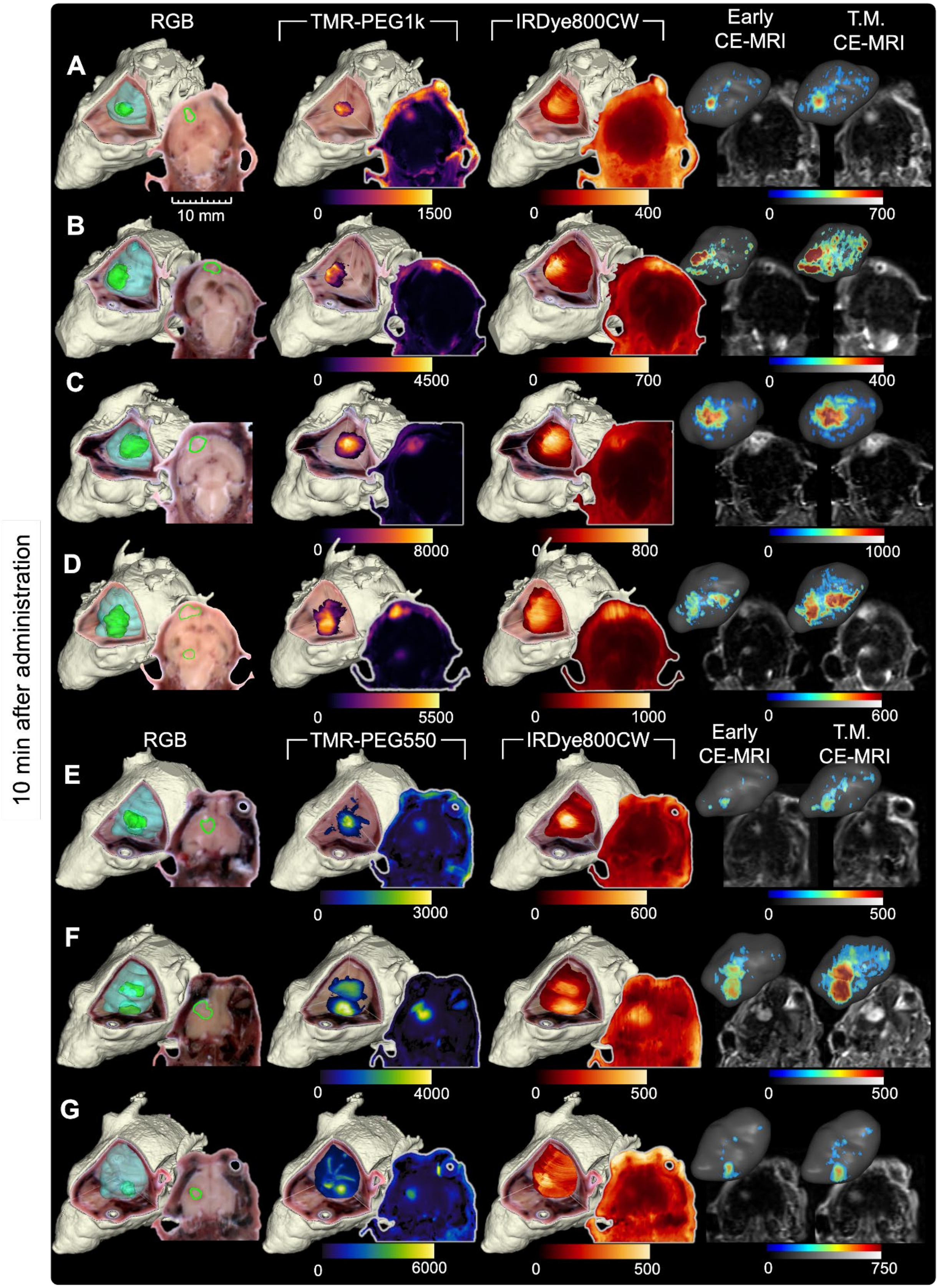

RGB
TMR-PEG1k
IRDye800CW
Early CE-MRI
T.M. CE-MRI
A
10 mm
0
1500
0
400
0
700
B
0
4500
0
700
0
400
C
0
8000
0
800
0
1000
D
0
5500
0
1000
0
600
10 min after administration
RGB
TMR-PEG550
IRDye800CW
Early CE-MRI
T.M. CE-MRI
E
0
3000
0
600
0
500
F
0
4000
0
500
0
500
G
0
6000
0
500
0
750

Figure S1. Cryo-imaging and CE-MRI image volumes of the remaining animals with U251 tumors 10 min after agent administration (described in Figure 2).

Figure S2.

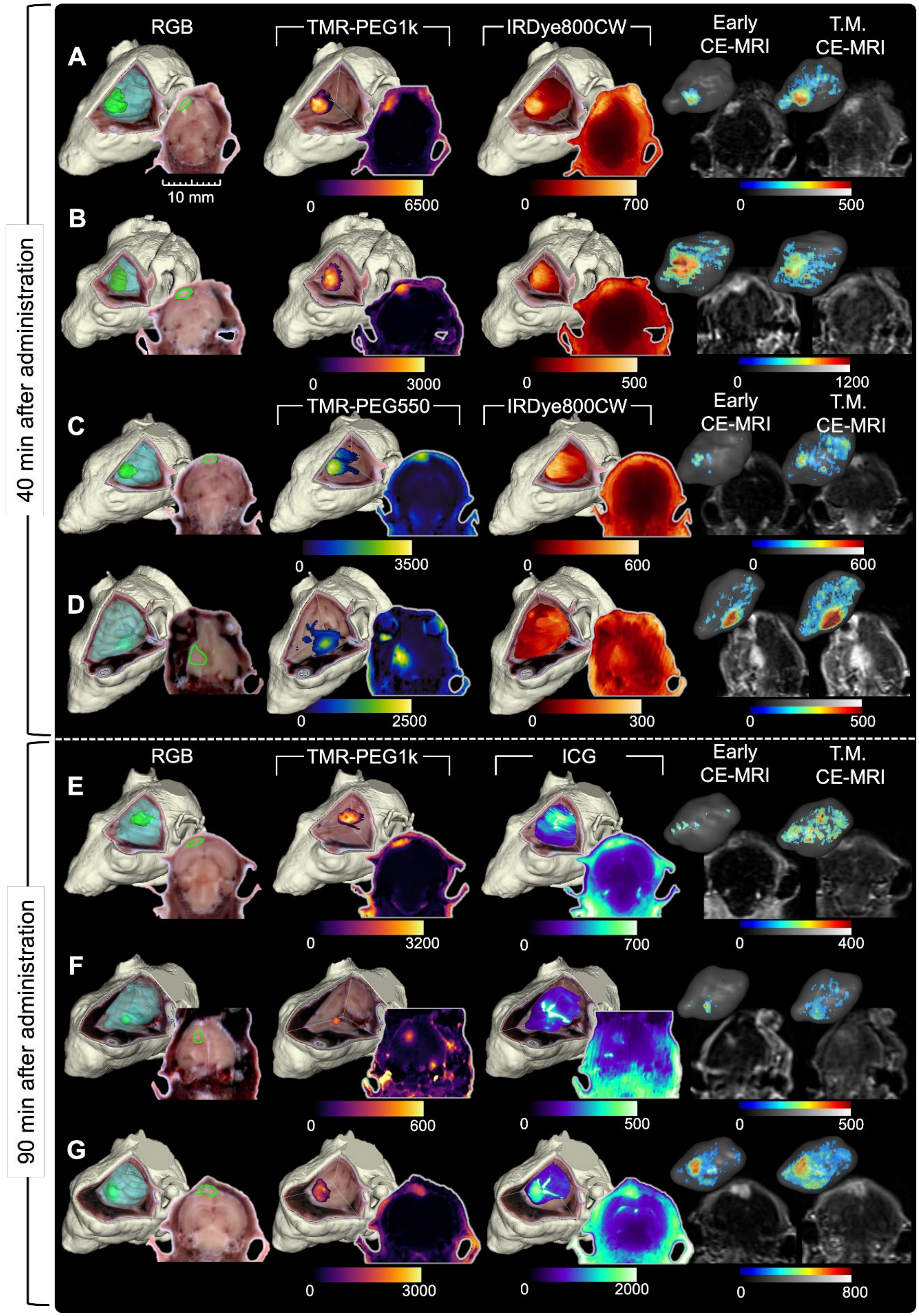

40 min after administration
90 min after administration
RGB
TMR-PEG1k
IRDye800CW
Early CE-MRI
T.M. CE-MRI
A
10 mm
0
6500
0
700
0
500
B
0
3000
0
500
0
1200
TMR-PEG550
IRDye800CW
Early CE-MRI
T.M. CE-MRI
C
0
3500
0
600
0
600
D
0
2500
0
300
0
500
RGB
TMR-PEG1k
ICG
Early CE-MRI
T.M. CE-MRI
E
0
3200
0
700
0
400
F
0
600
0
500
0
500
G
0
3000
0
2000
0
800

Figure S2. Cryo-imaging and CE-MRI image volumes of the remaining animals with U251 tumors 40 and 90 min after agent administration (described in Figure 3).

Figure S3.

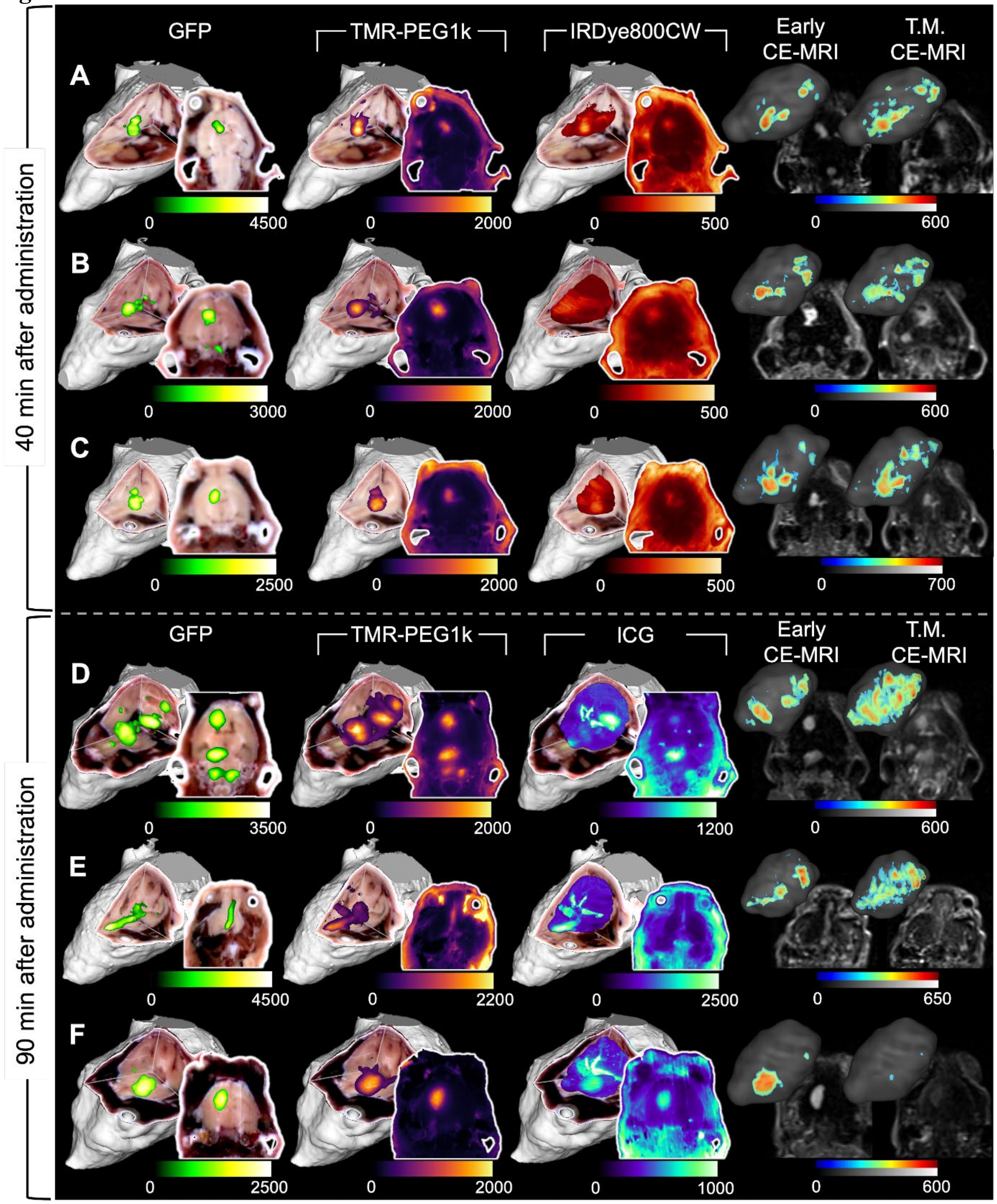

Figure S3. Cryo-imaging and CE-MRI image volumes of the remaining animals with U87 tumors 40 and 90 min after agent administration (described in Figure 6).

Figure S4.

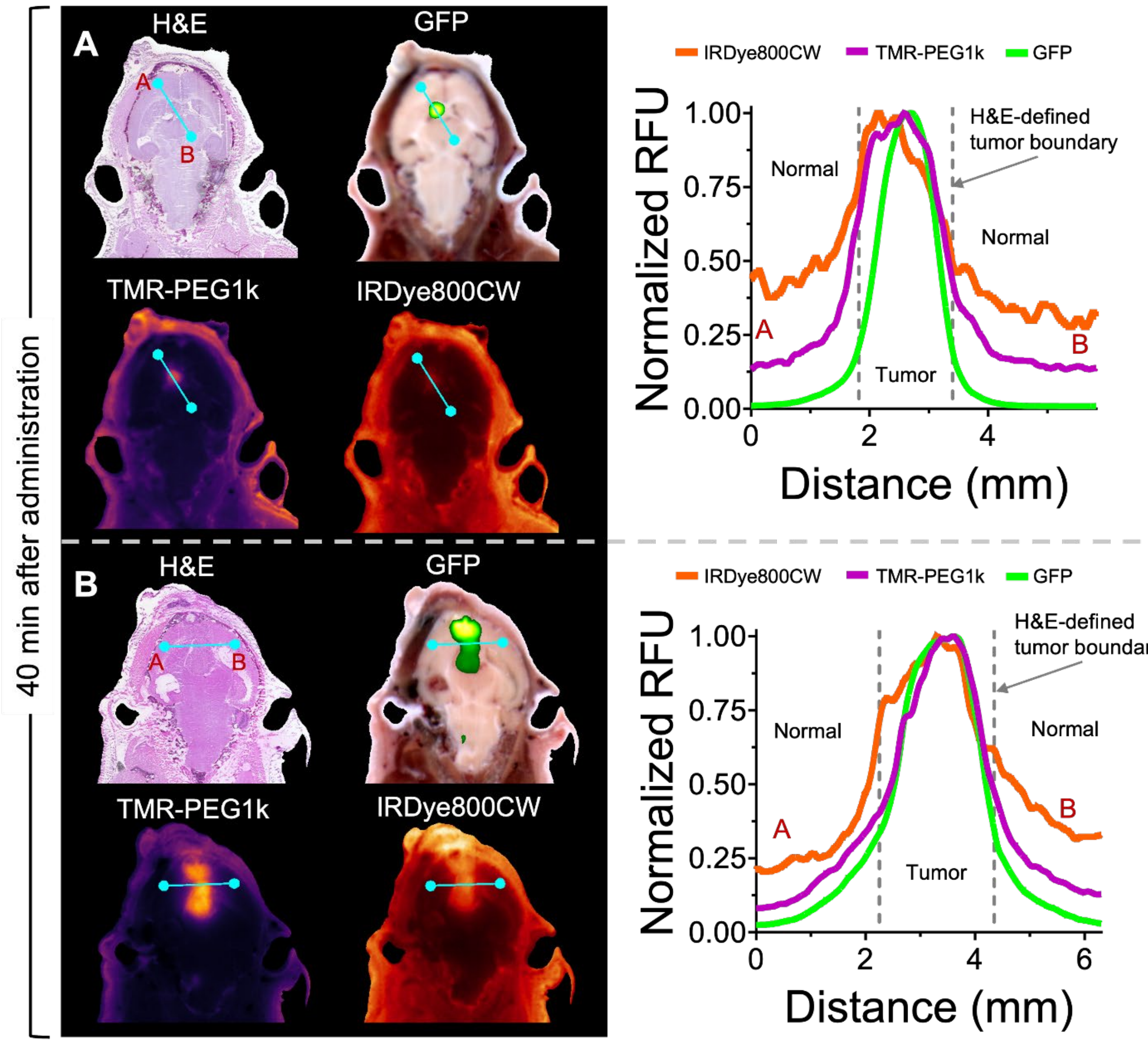

Figure S4. Co-registered H&E, GFP and contrast agent fluorescence for one section, and normalized fluorescence intensity profiles of GFP and contrast agent fluorescence for two animals (**A-B**) with U87 tumors 40 minutes after agent administration (described in Figure 8E-I).

Figure S5.

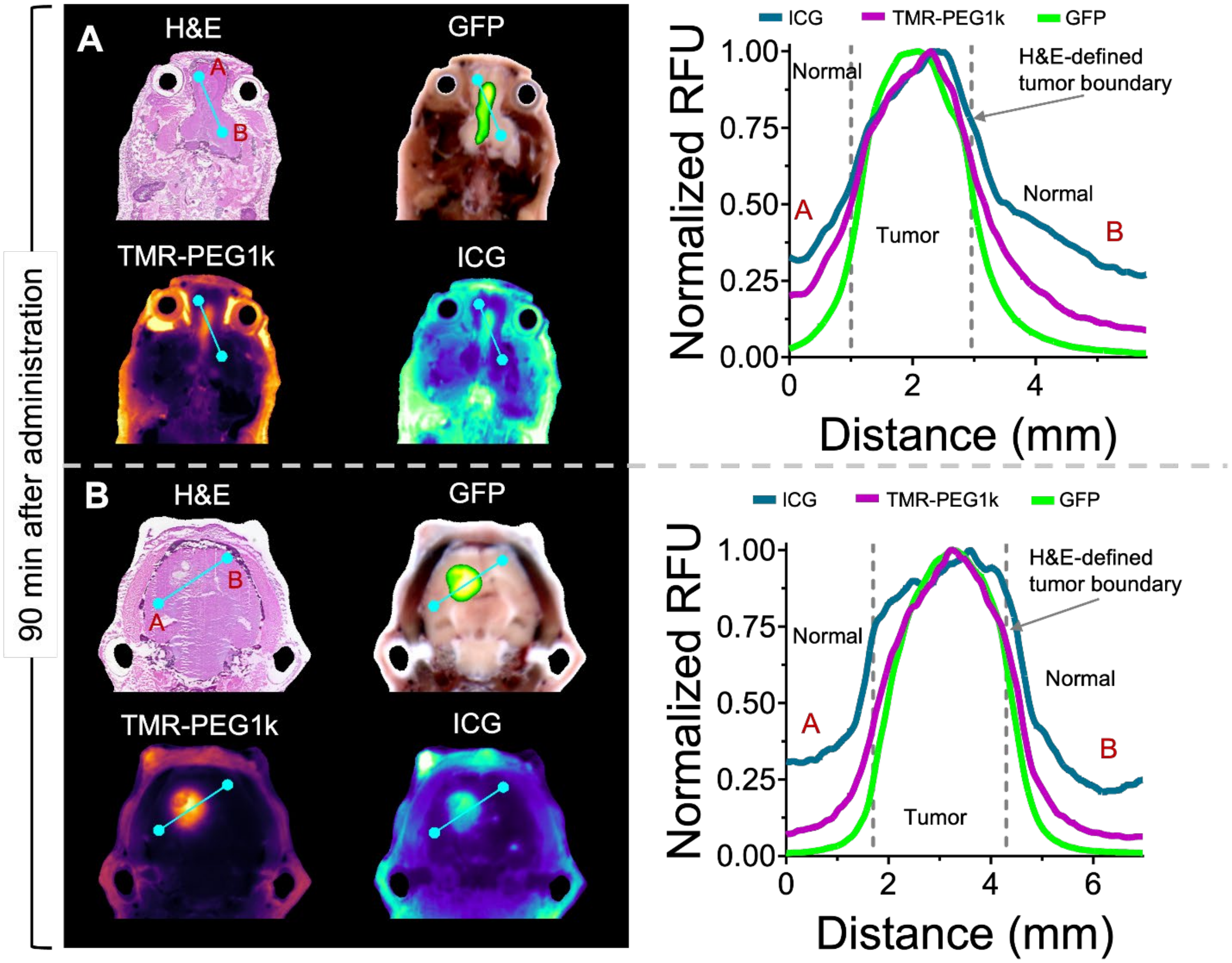

Figure S5. Co-registered H&E, GFP and contrast agent fluorescence for one section, and normalized fluorescence intensity profiles of GFP and contrast agent fluorescence for two animals (**A**-**B**) with U87 tumors 90 minutes after agent administration (described in Figure 8E-I).

Table S1.

Table S1. P-values for comparing TBR and CNR for paired fluorescent contrast agents injected in the same animal.

Paired TBR Contrast Agents	10 min	40 min	90 min
TMR-PEG1k vs. IRDye800CW	0.002**	0.028*	---
TMR-PEG1k vs. ICG	---	---	0.020*
TMR-PEG550 vs. IRDye800CW	0.132ns	0.174ns	---
**Paired CNR Contrast Agents**			
TMR-PEG1k vs. IRDye800CW	0.040*	0.078ns	---
TMR-PEG1k vs. ICG	---	---	0.040*
TMR-PEG550 vs. IRDye800CW	0.089ns	0.125ns	---

Note: $^{****}p \leq 0.0001$, $^{***}p \leq 0.001$, $^{**}p \leq 0.01$, $^{*}p \leq 0.05$, $^{ns}p \geq 0.05$.
